# Vat Photopolymerization of Smart Polymers for Biomedical Applications

**DOI:** 10.1021/acsapm.5c01487

**Published:** 2025-09-04

**Authors:** Syed Muhammad Zubair Shah Bukhari, M. A. Bukhari, Mokarram Hossain

**Affiliations:** † 50121 IMT School for Advanced Studies Lucca, Piazza San Francesco 19, Lucca 55100, Italy; ‡ Department of Physics, The University of Lahore, Lahore 54000, Pakistan; § Zienkiewicz Institute for Modelling, Data and AI, Faculty of Science and Engineering, Swansea University, Swansea SA1 8EN, U.K.

**Keywords:** vat photopolymerization, smart polymers, soft robotics, tissue engineering, drug delivery, biomedical devices

## Abstract

Additive manufacturing techniques, particularly vat photopolymerization (VPP), have emerged as significant drivers of advancements in materials and technology. VPP offers unparalleled precision and detail in translating complex three-dimensional (3D) forms, making it particularly suitable for smart polymers responsive to external factors such as pH, heat, magnetic fields, electric fields, humidity, light, and temperature. This review comprehensively explores the mechanisms and applications of VPP in fabricating smart polymer-based structures for biomedical purposes. It begins by detailing various VPP methods, highlighting the growing demand for innovative solutions in the biomedical sector. The review further examines the advantages of VPP, including its capability to handle intricate geometries, facilitate rapid prototyping, and provide design flexibility with diverse material options. Additionally, it discusses the challenges and prospects of materials such as bioabsorbable polymers and bioinks, emphasizing their role in bone tissue engineering, dentistry, drug delivery, and tissue regeneration. This review could be a valuable resource for biomedical engineers and clinical researchers seeking to integrate advanced printing technologies into biomedical applications.

## Graphical abstract

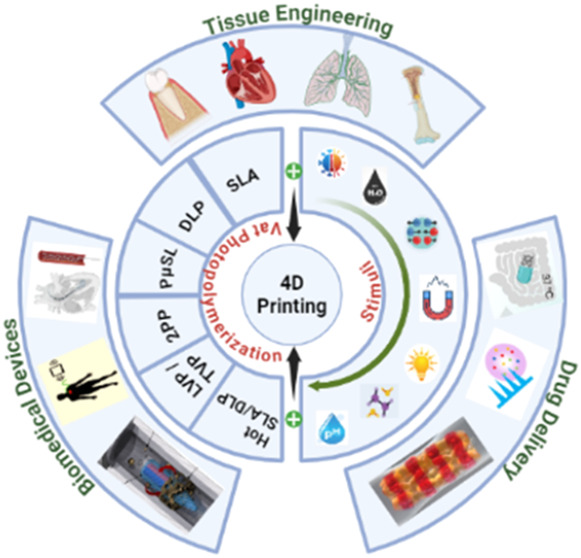

## 1 Introduction

The manufacturing industry is experiencing a surge in the adoption of innovative techniques and methods aimed at enhancing productivity, sustainability, and cost-efficiency. Traditional manufacturing processes primarily involve material removal, whereas contemporary approaches emphasize the addition of the requisite materials to fabricate intricate geometric objects. This additive manufacturing (AM) process, also called three-dimensional (3D) printing, had its origins in the early 1980s and has since undergone continuous developments and refinements, particularly in an array of material types and printing processes.^1^ Notably, 3D printing technologies find applications in aerospace, biomedical, automotive, dental, food production, and even the construction sector.^2–6^ Various 3D printing techniques are available, and the choice depends on the materials and specific requirements. Notable methods include vat photopolymerization (VPP), binder jetting, powder bed fusion, material extrusion, material jetting, directed energy deposition, and sheet lamination.^7–11^ Material jetting employs inkjet technology, where an inkjet head moves in Cartesian coordinates to deposit, build, and support materials.^12^ In binder jetting, powdered material is consolidated into a 3D shape using a binder material.^13^ Material extrusion utilizes controlled deposition of heated filament material through a nozzle to create desired shapes.^14^ Powder bed fusion, notably in the form of laser powder bed fusion, uses a bed of powdered material onto which a laser or electron beam is directed to fuse the material into a desired shape, making it a common choice for metal printing.^15^ Sheet lamination printing is characterized by the construction of objects through the layering and bonding of thin sheets of material, often utilized in the production of large, lightweight components.^16^ Directed energy deposition (DED) is another AM process that uses a focused energy source to melt and deposit material.^17^ VPP, for instance, relies on liquid resins and ultraviolet (UV) light to solidify materials, forming 3D objects.^18^

VPP consists of various processes, including stereolithography (SLA),^19^ digital light processing (DLP),^20^ continuous liquid interface production (CLIP),^21,22^ hot lithography/DLP (hot SLA/DLP),^23,24^ tomographic/linear volume printing (TVP/LVP),^25–27^ and two-photon polymerization (2PP).^28^ VPP stands out as a highly precise and high-resolution technique, with a layer thickness as fine as 1 μm, in stark contrast to fused deposition modeling (FDM), which typically features a 100 μm layer thickness.^29^ This fine layer resolution in the VPP enables the fabrication of intricate internal structures.^30^ Furthermore, VPP exhibits superior production speed in comparison to other 3D printing methods that makes it a competitive choice for heavy workloads.^31^ The platform scalability of VPP allows for the simultaneous fabrication of multiple objects, enhancing its efficiency. Additionally, these fabricated parts can possess both flexible and rigid characteristics, while minimizing material waste, as uncured resins can be continuously utilized in the process.^32–34^

VPP technology enables adoption in a diverse sectors of industries, including jewelry,^35^ low-run injection molding, water filtration, and automotive industry,^36^ dental, and various medical fields.^37^ Within the realm of medical applications, VPP proves invaluable for creating highly complex porous structural geometries in bone-related applications,^38^ regenerative medicine,^39–42^ scaffolds,^43–45^ tissue engineering (TE),^46–48^ and the development of medical devices.^49,50^ It has a major impact on dentistry,^51^ orthopedics,^52^ implantology,^53^ the production of surgical tools,^54^ and the development of special-purpose medical apparatuses,^55^ among other critical medical applications. In numerous biomedical applications, there is a demand for dynamic objects that demonstrates adaptive capacity over time, surpassing the capabilities of static structures such as 3D-printed tissue or organ.^56–58^ This requirement goes beyond the capabilities of traditional 3D printing methods,^59^ compelling the evolution of more advanced technology solution to meet functional dynamic needs.^60^

To meet these requirements, the so-called four-dimensional (4D) printing has been introduced. 4D printing is an intriguing extension of 3D printing, where fabricated objects undergo predetermined transformations in response to external stimuli.^61^ This innovative approach allows 3D-printed objects to exhibit changes in their shape, structure, and functionality over time,^62^ introducing dynamic properties to the material and design landscape.^63^ The external stimuli that drive these transformations in printed objects can encompass a range of factors, including light,^64,65^ temperature,^66^ pH levels,^67^ electrical impulses,^68^ magnetic fields,^69,70^ humidity,^71,72^ and even cellular traction forces.^73^ This dynamic quality opens numerous possibilities for applications in various fields, as illustrated in Figure 1. VPP techniques play a pivotal role in enabling 4D printing by facilitating the use of smart polymers, along with liquid crystal elastomers (LCEs), active hydrogels, and shape memory polymers (SMPs).^74,75^ These advanced multifunctional polymers can undergo controlled and reversible transformations in response to external stimuli, further enhancing the potential of 4D printing.^76^ The applications for 4D printing are diverse and promising. It spans a wide spectrum of medical field, including drug delivery, where dynamically changing drug release profiles can be achieved,^77^ stents that adapt to the specific requirements of a patient’s blood vessels, shape-memory resins for AI-aided personalized to fabricate scaffold,^78^ soft robotics that exhibit adaptive behavior and locomotion,^79^ microfluidics for precise fluid control and manipulation, and heterogeneous constructs for TE,^80^ where the dynamic properties of printed constructs offer new possibilities for regenerative medicine.^81^

**Figure 1:**
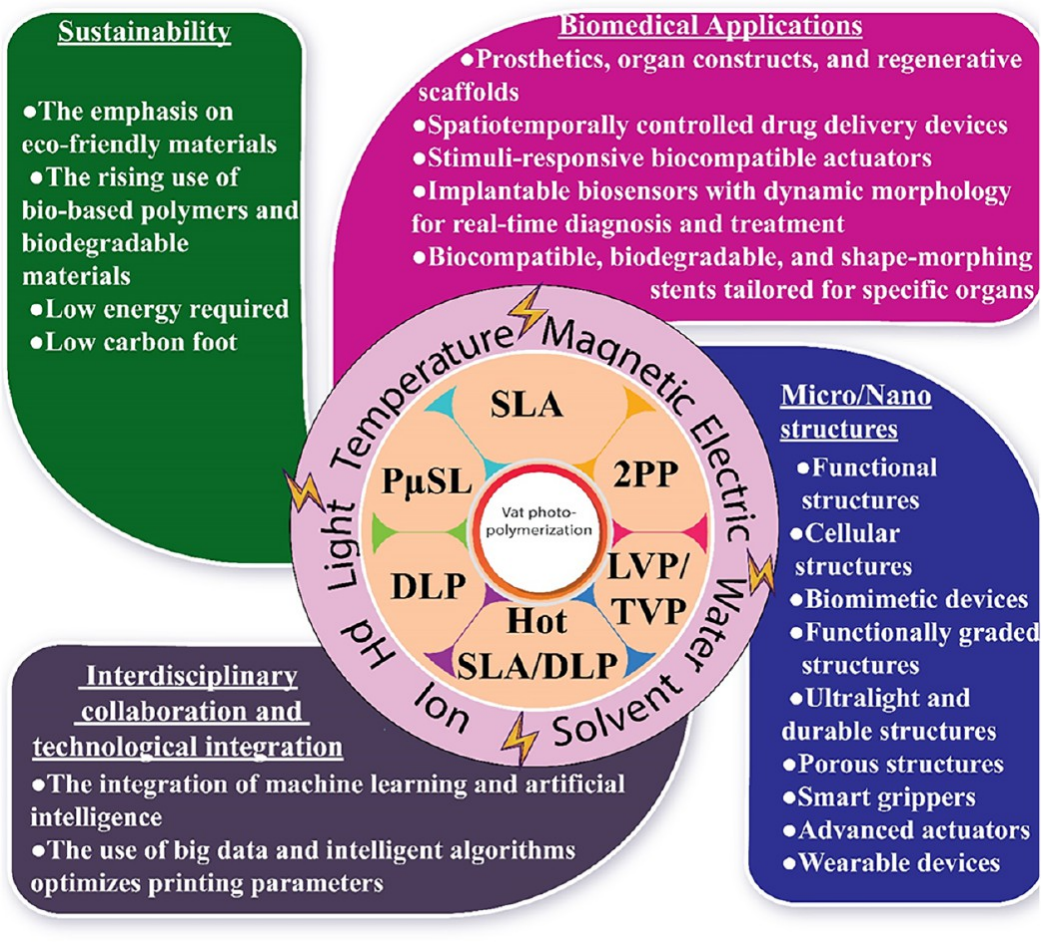
Use of VPP technology in different sectors.

Although 4D printing has made considerable progress and shows great promise, there are still critical limitations remaining to be addressed in terms of choosing suitable materials, achieving precise printing, and making these technologies scalable for widespread use in clinical and industrial settings. This review is to offer a thorough examination of the status of VPP printing technology, its uses in the biomedical sector, and the current challenges. Through a thorough analysis of recent research and advancements, our aim is to emphasize the potential advantages and challenges that lie in the future, providing valuable insights into the transformative impact of VPP technology in the biomedical field.

## 2 Vat Photopolymerization

VPP process involves photosensitive materials that are exposed to light, causing them to transition from a liquid to a solid state in a layer-by-layer process. The vat, acting as a container, holds resins or photopolymerizable materials.^82^ Creating a 3D object begins with using a computer-aided design (CAD), which is later converted to an STL file format. The slicing of these files is accomplished using specialized software. The ensuing instructions are under the precise control of the computer, guiding the adjustment of the laser beam’s direction, location, and the platform’s positioning. Subsequently, these layers are printed to construct a 3D object, with specific materials such as photopolymers or resins^83^ being essential for the printing process.^84^ VPP bioprinting is making an extreme impact in biomedical applications like fabricating microtissue models for drug discovery and regenerative medicine.^85,86^ Advancements in biomaterials have paved the way for the effective integration of living cells and bioactive agents into tailor-made printable bioinks, specifically used for VPP applications.^87–89^ Various curing sources are employed in printing, leading to the categorization of this technique into different types, including SLA, DLP, PμSL, 2PP, hot SLA/DLP, and TVP/LVP, as illustrated in Figure 2. Table 1 summarizes the different VPP printing methods and their advantages, disadvantages, and biomedical applications.

**Table 1:** Different VPP Processes, Their Advantages, Disadvantages, and Biomedical Applications

**types**	**material(s)**	**function**	**advantages**	**disadvantages**	**biomedical application(s)**	**refs**
**SLA**	ABS, PLA, OMA-PEGDA, GelMA and HAp, epoxy resin, polyester resin, and photosensitive polyacrylate resin	• liquid resin polymerized by UV or visible light associated with raster laser scanning and transformed into a solid object.	i. intricate geometry shape	i. laser is a single point, so it is time-consuming compared to others.	i. dental and orthodontics	^95–99^
		• bottom-up (laser setup is under the transparent window of resin bath) more affordable and advantage of small volume fabrication.	ii. better surface finish as compared to traditional manufacturing.		ii. orthopedics implants	
		• top-down (the laser setup is above the resin)	iii. high resolution and fine details		iii. drug delivery	
		• resolution from 75 to 200 μm with printing area 62,500–14,70,000 mm^2^.			iv. prosthetics	
					v. surgical planning	
**DLP**	natural	• 2D photopolymerization using DMD or LCD projection system.	i. sophisticated geometry and surface finish.	i. bioink must photo-cross-link easily under light irradiation.	i. TE	^100–102^
	• GelMA	• DMD is an array of millions of micromirrors that (switches on and off) only transform light when it is on.	ii. time saving and resin saving (only in the bottom-up approach)	ii. bioink viscosity needs to be relatively low for continuous interfacing with cured layers.	ii. regenerative medicine	
	• GelAGE	• it is two types bottom-up (using transparent glass made of thin-layer polydimethylsiloxane (PDMS) or Teflon membrane) and top-down.		iii. difficulty in achieving truly volumetric structures.		
	• HAMA	• home theater projectors (1920 × 1080 pixels) and optical engines (1280 × 800 or 1920 × 1080 pixels) with DMD chips are commonly employed in DLP-based 3D bioprinting, offering an xy-resolution of 10 μm and a projection area of 96 × 54 mm.		iv. DLP-based bioprinting typically restricted to planar structures.		
	• dECM	• resolution to area respectively (7.6–65 with 234.135- 8760.96)		v. challenges in bioprinting with ultrasoft bioinks due to the potential for deformation or collapse during layer-by-layer printing.		
	• Sil-MA					
	synthetic					
	• PEGDA					
	• PVA-MA					
	• PGSM					
	GelMA and HAMA, Silk-GMA, GelMA/PEGDA					
**PμSL**	poly(ethylene glycol) diacrylate (PEGDA) and 1,6-hexanediol diacrylate (HDDA), e acrylamide-PEGDA hydrogel	• a 2D CAD pattern is projected onto a DMD, which emits UV light. this light passes through a reduction lens and focuses on resin, causing localized photopolymerization.	i. good surface finish	i. build platform and transparent constrained window are still required.	i. drug screening	^103–107^
		• resolution (0.6–30 μm) with printing area (2–768 mm^2^)	ii. solidify photopolymerization resin at once with microdetails		ii. disease study	
					iii. TE	
					iv. central nervous system	
					v. regeneration, and cell-seeding scaffolds.	
**2PP**	• photoresist ((SU-8, IP-L), (SU-8, PEG), (SU-8, ferrofluid), (IP-S, IP-Visio), (FemtoBond 4B))	• ultrafast laser to selectively polymerize a liquid resin which achieves submicrometer resolution.	i. high resolution and precision	i. long printing time for millimeter-scale structures due to submicron-size voxels	i. cell engineering	^108–110^
	• SMP ((HPPA, BPA, TPO), (IsobA, PEGDA 575, TcddA), (benzyl methacrylate-based SMP), (AAc, HPPA, PVP, DPEPA))	• the quadratic intensity dependence of 2PP facilitates tight laser confinement, permitting voxel generation with micron-scale precision	ii. material versatility	ii. inevitable connection gaps in final samples using voxel-to-voxel printing method.	ii. TE	
	• hydrogel (PEGDA, Irgacure 369, (GelMA, lithium phenyl(2,4,6-trimethylbenzoyl) phosphinate, iron oxide), (PEGDA, PETA), (PEGDA, MB))	• resolution to area relationship (0.08–0.16 μm with area 0.0064–12.25 mm2)		iii. limited biomaterial choices for 3D soft tissue scaffolds	iii. medical devices	
	• LCE ((Difunctional and monofunctional mesogenic acrylates, photoinitiator), (ST3021, ST3866, irgacure 369, dye DR1A))			iv. low spatial resolution in 3D hydrogel scaffolds prepared by 2PP.	iv. bioinspired microactuators	
				v. difficulty achieving precise manipulation of nanoscale topography and pore dimensions	v. biomedical microrobots	
					vi. repellent and antiadhesive structures for medical implants and antimicrobiotic surfaces	
					vii. Microneedles for drug/vaccine delivery	
**hot SLA/DLP**	• PCL, PPG–PCL-DA, PGS-A, PPF, PGD-A, PEG–PU-DA	• resolution 30–100	i. accelerates curing time	i. require precise temperature control	i. vascular grafts	^24^
		• viscosity 10 at 80–100	ii. extended from room temperature printing	ii. limited speed	ii. liver lobul	
			iii. reduce resin viscosity		iii. scaffolds	
					iv. tracheal stents	
**linear volumetric printing**	• BSA-PEG-A, BSA, PEGDA, TMPAC-*co*-NTC	• resolution 25 μm	i. high resolution	i. transparent resin required	TE	^26,94,111^
		• viscosity 10–80	ii. rapid fabrication	ii. highly viscous resins	drug delivery	
			iii. uniform mechanical properties	iii. limited size		
			iv. enhanced surface quality	iv. specialized equipment requirements		
**tomographic**	• PCL, PLA	• resolution 80–300	i. high speed and resolution	i. high transparency	i. dental devices	^112,113^
**volumetric printing**		• viscosity 4–93	ii. complex internal structures	ii. limited size	ii. drug delivery	
			iii. uniform curing	iii. scale limitation	iii. scaffolds	
				iv. complexity in light control		

**Figure 2:**
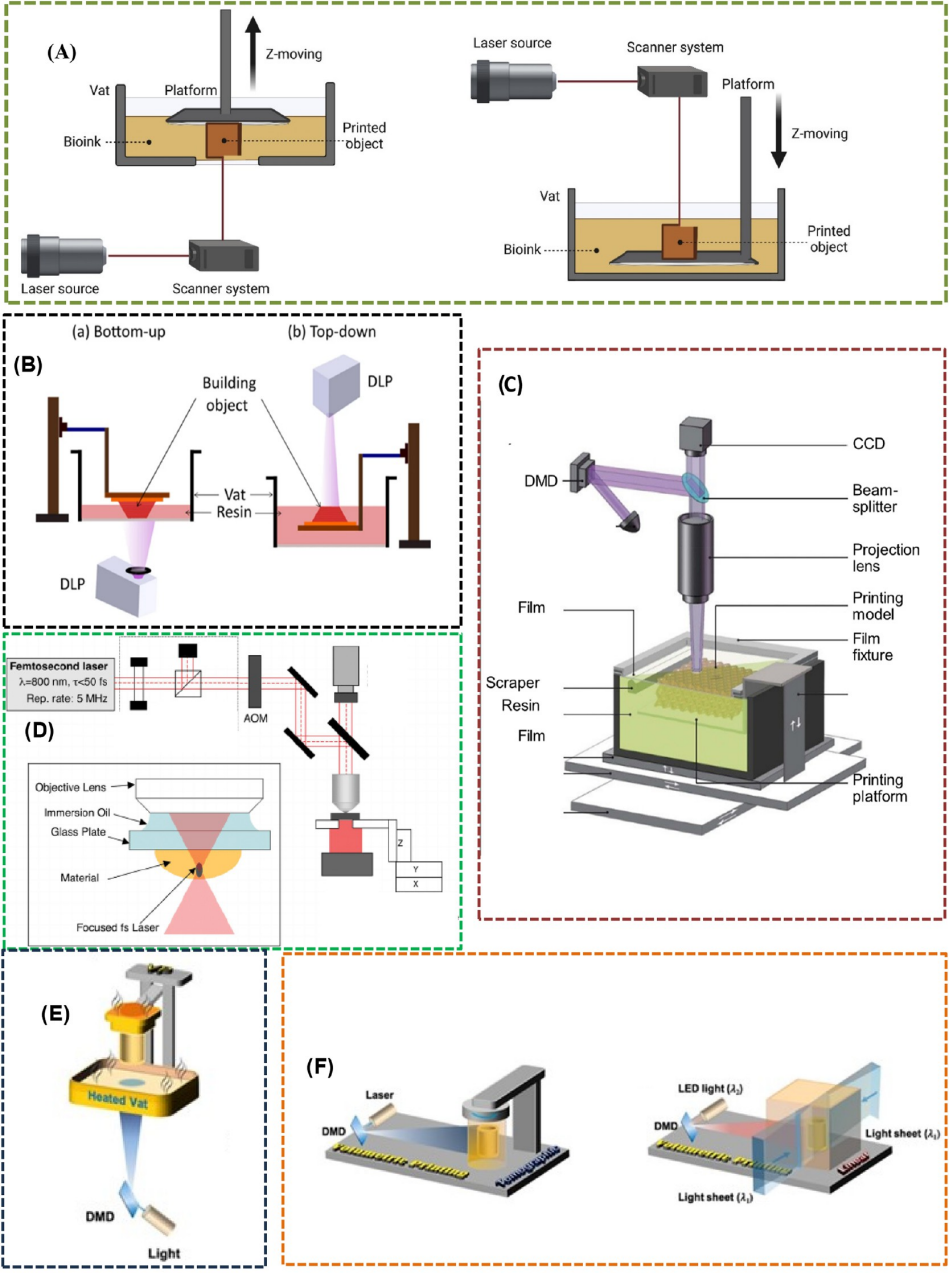
Schematics illustration of VPP processes (A) SLA (adapted from ref 90, under the creative commons CC-BY-NC-ND license); (B) DLP (adapted from ref 91, under a creative commons attribution 4.0 license); (C) PuSL (adapted from ref 92, under the terms of the creative commons attribution 3.0 license); (D) 2PP (adapted from ref 93, under the terms of the creative commons CC-BY license); (E) Hot Lithography/DLP (Reproduced under the terms of the Creative Commons CC BY license from ref 94 Copyright 2024); (F) Tomographic (left) and Linear (right) volumetric printing (xolography). (Reproduced under the terms of the Creative Commons CC BY license from ref 94 Copyright 2024).

### 2.1 Stereolithography

SLA is one of the most prevalent methods in additive manufacturing, employing a laser beam that traverses a scanned system before reaching the liquid photosensitive polymer,^90^ as illustrated in Figure 2A. Curing involves gelation (liquid to rubber, irreversible) and vitrification (rubber to glass, thermoreversible) via cross-linking. Photoinitiators (0.5–12 wt %, radical/cationic) control curing dept (0.1–2.5 mm) based on efficiency, solubility, and stability.^114^

SLA plays a crucial role in biomedical applications, particularly in the integration of patient-specific manufacturing for implantable devices, prosthetics, surgical guides and tools, and preoperative planning models. Traditional manufacturing methods often fall short in achieving the level of precision required for such patient-specific applications. SLA is leveraged to craft patient-specific physical models, encompassing soft, hard tissues, and even introducing color to distinguish pathological components.^115^ This application aids clinicians in comprehending complex anatomies, facilitating informed decision-making for surgical interventions, and predicting postsurgical outcomes. The customization process involves the utilization of a DMD-based rapid manufacturing technique for mass customization of patient-specific ears.^116^ Biocompatibility of implants is a critical factor, as they come in direct contact with native and regenerative tissues or organs, unlike prosthetics. Given that resins in SLA possess toxicity that can be harmful to tissues, it is imperative to utilize biocompatible materials. Achieving biocompatibility in SLA photosensitive material involves treating it with supercritical carbon dioxide to eliminate potential toxic residues.^117^ Bioactive, biodegradable, and biocompatible materials play a vital role in TE by utilizing cells and biomaterials for printing. These resins act as supportive guides, fostering a supportive milieu for the growth and formation of new tissue.^118^ The demand for tissue, currently met by autografts and allografts, falls short due to low supply and inability to meet the complexity of requirements.^119^ SLA addresses this gap with biocompatible and biodegradable materials, utilizing polymer-ceramics for scaffolds as a cell-seeding platform.^120^ Trileaflet heart valve scaffolds were crafted using poly-4-hydroxybutyrate and polyhydroxyoctanoate, placed in specific conditions to replicate in vitro opening and closing behavior.^121^ SLA technique involves printing various polymeric materials, such as photocurable resins or photo-cross-linkable hydrogels, to fabricate parts capable of changing their shapes. The biomedical applications discussed above represent only a subset of SLA fabrication’s extensive utilization within the field, highlighting its prevalent use in biomedical manufacturing.

### 2.2 Digital Light Processing

DLP utilizes light to polymerize liquid resin into solid form.^122^ Employing the digital micromirror device method,^123^ 2D images of an object are projected onto the uncured resin layer, with micron-sized mirrors determining the XY-plane resolution,^124^ as illustrated in Figure 2B. Two geometrical approaches,^125^ top-down and bottom-up, exist in the DLP, each with its advantages and drawbacks. In the top-down method, the platform descends, projecting a 2D image layer onto the resin layer. Polymerization occurs upon exposure to light, and as the platform moves down, the next uncured layer rises with the desired thickness. This iterative process continues until the desired object shape is achieved. Conversely, the bottom-up technique projects light from the bottom, with the glass curing the resin upward with a layer-by-layer process. This method eliminates the need for recoating, and the vacuum ensures autofilling, even with viscous resins. Challenges in bottom-up include adhesion between the vat bottom and polymerized resin, addressed through flexible films, coated films,^22,126^ and separation movements.^127,128^ While top-down method avoids adhesion issues, environmental oxygen on the resin surface can hinder the polymerization process.

DLP finds application in diverse biomedical contexts, replicating the shapes of organs such as the ear, brain, heart, trachea, lungs, and vascular structures.^104^ In the realm of material studies, 3D-printed zirconia implants are created using DLP to analyze mechanical properties, surface topology, and dimensional accuracy.^129^ Biomedical applications extend to TE, employing bioink with essential qualities such as biocompatibility and biomechanical properties. The versatility of DLP is evident in the fabrication of bioactive ceramic scaffolds, exploring the impact of pore and structure size on mechanical performance.^130^ Utilizing glass-filled photosensitive resin in the production of bioactive glass-ceramic scaffolds^131^ and hydroxyapatite (HAp) scaffolds.^132^ DLP serves to assess biocompatibility with tissues. Beyond structural components, DLP contributes to microTE for the liver, employing cell-laden bioink.^133^ Furthermore, the application of DLP in bone repair involves the use of calcium phosphate (CaP). DLP-based process to bioprint artificial cartilage structures in vitro, using gelatin methacrylate (GelMA) or HAMA-based bioinks to mimic cartilage formation.^134^ This multifaceted utilization underscores the significance of DLP in advancing various aspects of biomedical research and applications.^91^

### 2.3 Projection Microstereolithography

PμSL, a technique employed for microscale manufacturing, utilizes a dynamic mask device (DMD).^107^ The process involves the fabrication of intricate 3D objects layer by layer,^92^ as illustrated in Figure 2C. The initial step includes slicing a CAD model into 2D layers to form an image of the object, which is then fed into the DMD for modulation. UV light, patterned according to the 2D object, passes through a reduction lens and falls onto the resin, curing the photosensitive material. The part is iteratively fabricated by this process. The initial PμSL demonstrated the capability to produce microsprings and threads with a size of approximately 0.6 μm. Key factors influencing this technique include the photoabsorber and optical intensity. Commercially available PμSL systems exhibit a resolution of 2 μm per pixel within a 50 × 50 mm area and 10 μm within a 94 × 3 mm printing area. The performance is improved by using the liquid crystal on silicon chip as dynamic mask and for light source UV LED is used.^135^ In the realm of TE using SLA-based methods, pioneering studies have utilized direct laser writing and conventional UV irradiation for polymerization.

In the realm of biomedicine, application involves fabricating TE scaffolds or living constructs with biomaterials and advanced techniques. This advancement allows for a more accurate replication of the native microenvironment, enhancing cellular behaviors and functions. To address the limited cell adhesion properties observed in conventional UV-curable hydrogels such as PEGDA and the combination of PEGDA with GelMA, an effort was made to improve the cell affinity using silk fibroin (SF), a naturally fibrous protein. A UV-curable bioink was developed with SF to ensure compatibility with PμSL.^104^ The resulting Sil-MA bioink proves to be well suited for tissue and organ engineering using PμSL. In the realm of biomedical devices, diverse applications highlight the versatility of PμSL. Notably, tilted microneedles have been developed for efficient drug injection, providing a novel approach to targeted drug delivery. Microstructures such as the micro buckyball find application in cell cultivation, contributing to advancements in cell culture techniques. Another innovative application involves the creation of drainage nails designed for glaucoma treatment, demonstrating the adaptability of PμSL in addressing specific medical needs. These developments underscore the potential of PμSL in advancing precision medicine and tailored solutions for various biomedical challenges.^136^ In exploring fluid transport mechanisms within solid organs, characterized by intricate biophysical and biochemical vascular networks, PμSL was utilized to fabricate intravascular and multivascular structures. This involved incorporating food dye additives into photopolymerizable hydrogels, serving as biocompatible photoabsorbers. Such multifaceted approach contributes to the advancement of biomedical applications, particularly in the fabrication of TE scaffolds and the exploration of fluid transport in solid organs.^137,138^

### 2.4 Two-Photon Polymerization

2PP distinguished by its exceptional precision at the nanoscale.^139^ This process involves the use of a femtosecond laser to irradiate a liquid resin containing a photosensitive polymer,^93^ as illustrated in Figure 2D. The key innovation lies in the mechanism of two-photon absorption, where the material polymerizes upon the simultaneous absorption of two photons.^140^ This unique property enables highly localized and controlled solidification of the resin, occurring voxel by voxel at the focal point of the laser beam. This meticulous layering process ensures the creation of intricate and finely detailed structures. The unparalleled resolution of 2PP makes it particularly valuable in applications demanding microscale and nanoscale precision, such as microelectronics, micro-optics, 3D vascular microcapillary structures,^141^ and biomedical devices. Additionally, the two-photon method surpasses these capabilities by introducing biocompatible fabrication mechanisms with submicron resolution.^142^

The application of 2PP in medical contexts is notably evident in bone TE, where photosensitive materials, specifically methacrylamide-modified gelatin derived from native collagen, share chemical properties with cells.^143^ This choice of material aligns with the biocompatibility required for successful TE applications. Additionally, 2PP has been used in the fabrication of scaffolds for liver TE, using PLA-based structures.^144^ Furthermore, the technique has found utility in the creation of neural tissue scaffolds.^145^ The outcomes demonstrated that within the fabricated scaffolds, effective nutrient transport had been achieved, validating the cells’ ability to maintain their functionality. The versatility of 2PP extends to the fabrication of microneedles, spanning applications in micro-optics, photonics, and microfluidic systems.^146^ This diverse range of applications underscores the significance of 2PP in advancing various facets of medical research and technology. Table 2 presents literature studies that used VPP processes to develop multifunctional structures.

**Table 2:**
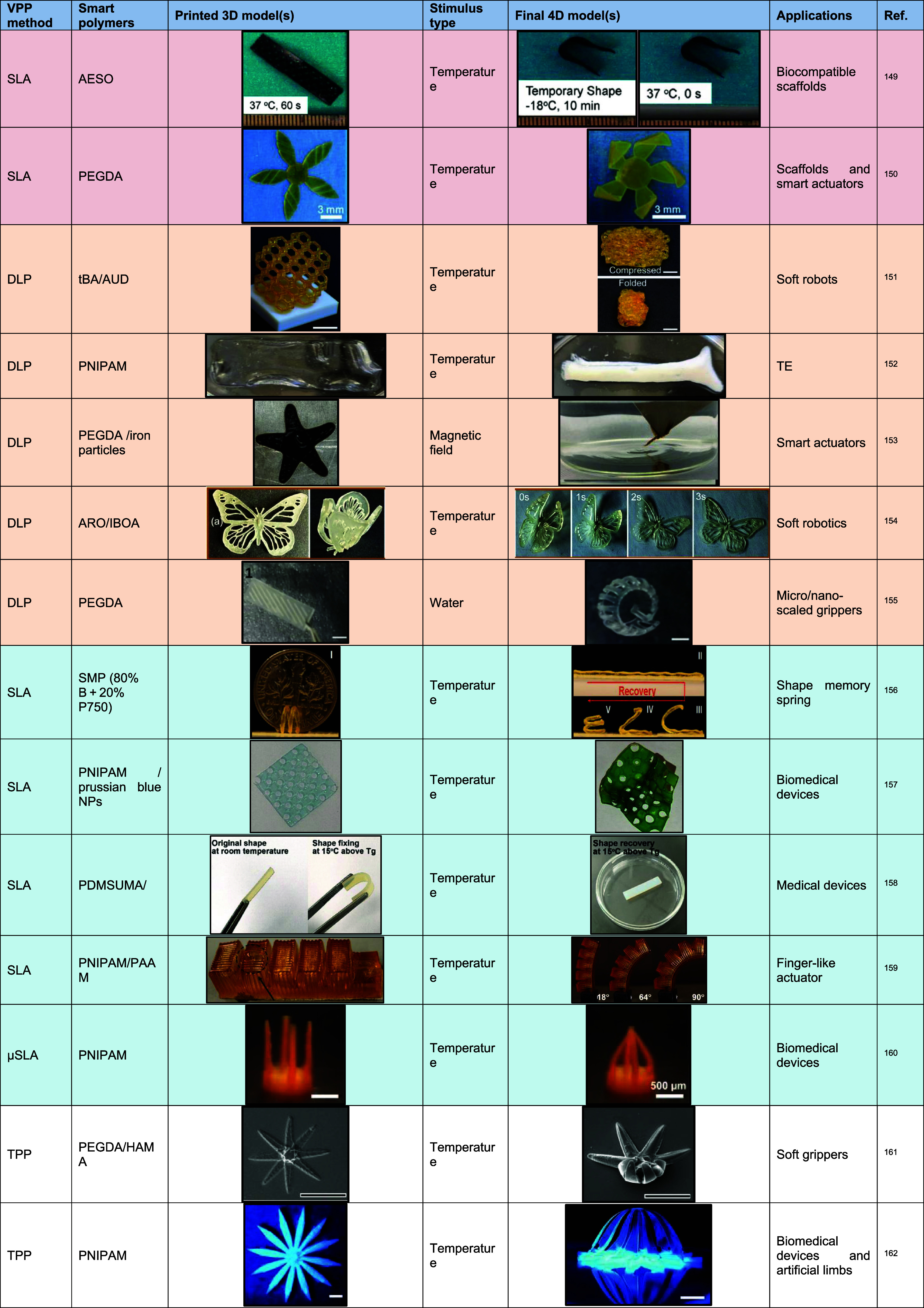
Summary of Literature Containing 3D-Printed Structures Developed for Biomedical Applications Using VPP Processes^149–162^

### 2.5 Hot Lithography/Heat-Assisted DLP

Hot lithography,^23,24^ or heat-assisted DLP,^147^ diverges from the conventional SLA/DLP processes by intentionally heating the resin during the printing. This approach enables the use of materials that are solid or highly viscous at room temperature such as biodegradable photopolymers such as PCL acrylates, which are otherwise unsuitable for standard vat photopolymerization. By elevating the resin temperature, the process effectively reduces the resin viscosity, thereby promoting better flow and more uniform layer formation as illustrated in Figure 2E. Enhanced thermal conditions accelerate the polymerization kinetics, leading to a notable reduction in cure times from approximately 50 s per layer down to 30 s while simultaneously improving energy efficiency. In terms of resolution, this method can achieve fine feature details, often in the tens of micrometers range, making it attractive for applications requiring high precision. However, the process demands rigorous temperature control to avoid thermal degradation and ensure consistent mechanical properties in the final product, and the integration of heating elements adds complexity and cost to the printing setup. Despite these limitations, the benefits of hot lithography, including expanded material compatibility and improved print performance, render it a compelling alternative for fabricating advanced biomedical devices and intricate microstructures.

### 2.6 Tomographic Volumetric Printing

Tomographic volumetric printing (TVP) enables the rapid fabrication of complex three-dimensional structures by simultaneously solidifying entire volumes of photosensitive resin using tomographic reconstruction principles,^25,27^ as illustrated in Figure 2F (left). Unlike traditional layer-by-layer methods, TVP projects dynamic light patterns from multiple angles, allowing for the creation of high-resolution objects (typically 80–300 μm) in a single step, significantly reducing the printing time, sometimes to mere seconds. This approach offers superior tolerance to resin viscosity (up to ∼93 Pa·s), expanding the range of compatible materials, including highly viscous biodegradable photopolymers. However, TVP currently faces limitations in scalability, as it is primarily suited for small-scale objects due to constraints in light penetration and resin transparency. Despite these challenges, its potential for high-speed, high-precision printing is promising for biomedical applications, such as personalized implants and dynamic scaffolds, where intricate geometries and rapid prototyping are critical. Further advancements in resin formulation and optical systems are expected to enhance their clinical translatability.

### 2.7 Linear Volumetric Printing

Linear volumetric printing (LVP)^26,148^ diverges from the traditional layer-by-layer methods by enabling the simultaneous solidification of entire volumes of photosensitive resin. This approach leverages tomographic reconstruction or intersecting beams of light to achieve rapid fabrication with printing speeds as fast as seconds and resolutions down to approximately 25 μm, as illustrated in Figure 2F (right). A key advantage of this method is its tolerance for highly viscous resins (up to ∼80 Pa·s), which expands the range of materials suitable for printing, including those with complex formulations. Despite its potential for high-resolution and efficient 4D printing, the technique is currently limited to small-scale objects, typically in the centimeter range, and requires resins with high optical transparency to ensure precise light penetration. While still in its early stages, linear volumetric printing holds promise for applications requiring intricate microstructures and dynamic, stimuli-responsive materials, though further development is needed to overcome its current constraints in scalability and material compatibility.

## 3 Biomedical Applications

3D printing technology has emerged as a pivotal force in biomedical applications, influencing the development of implants, organ printing, the pharmaceutical industry, prosthetics, TE constructs, drug delivery devices, dental applications, and stents, as illustrated in Figure 3. While traditional 3D printing techniques have primarily focused on creating static objects, there is a transformative shift toward employing smart polymers. These polymers exhibit the remarkable ability to dynamically change their shape, functionality, and programmability in response to specific requirements.^163^ This evolution in technology reflects the demand for adaptive materials that can perform tasks over time. The utilization of these advanced polymers in TE, drug delivery, and biomedical devices will be explored in subsequent sections, highlighting their diverse applications and transformative impact on healthcare technology.

**Figure 3:**
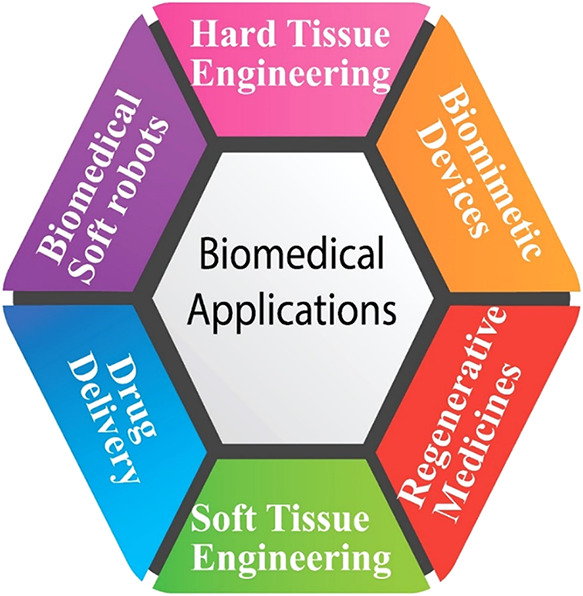
Different biomedical applications of VPP-printed structures.

### 3.1 Tissue Engineering

#### 3.1.1 Soft Tissue Engineering

Soft tissues encompass all of the tissues in a body except for bones and dental tissues. There are various types of soft tissues, including skin, cardiovascular, neural, gastrointestinal, liver, kidney, ocular, and musculoskeletal tissues. Each of these tissues serves distinct functions. They may be damaged due to injury and need to be replaced with surgical procedures. Replacing tissues with other materials poses significant challenges due to issues of biocompatibility and functionality. Although there are many methods for fabricating tissues, 3D printing enhances the ability to create shapes and geometries ranging from simple to complex. However, 3D printing has limitations, as it cannot produce artificial tissues that replicate dynamic characteristics. This is where 4D printing comes into play using biomaterials to create artificial tissues with dynamic properties. Mimicking the required tissue is essential, and biomaterials are used to fabricate soft tissues that can be used for replacements. The self-folding mechanism was employed to transform 2D planar geometry into 3D MHTs under the influence of external stimuli.^164^ This transformative process uses 4D printing achieved through the photopolymerization of shape memory hydrogels and MHTs to construct self-folding architectures.^165,166^ Hydrogels, responsive to water stimulation, facilitate self-folding, particularly in the creation of microvascular scaffolds.^167^ The implementation of 4D bioprinting involves GelMA/poly­(d,1-lactic acid) (PDLLA)-*co*-TMC-based scaffolds, wherein the regulation of orientation degree. Upon heating to physiological temperatures, these scaffolds autonomously fold, exhibiting diameters comparable to blood vessels.^168^

In the context of myocardial repair, 4D-printed cardiac scaffolds, incorporating polydopamine (PDA)/alginate and cell-laden hydrogel, are developed using NIR stimulus.^169^ These scaffolds prove highly conducive to cell culture by maintaining a deformed architecture. A bioengineered cardiac patch, fashioned from human endothelial cells, HMSCs, and HiPSC-CMs, is created by using SLA printing to fabricate a cellular patch. The myofibrils with different orientations used for myocardial infarction treatment,^170^ as illustrated in Figure 4A. The resultant 4D-printed patch enhances the blood density, biocompatibility, and attachment with cardiac tissue. Advancements in cardiac TE through 4D printing represent a paradigm shift, particularly in the treatment of patient-specific cardiovascular diseases. The development of tissue using HiPSC-CMs is stimulated by external stimuli, such as acoustics, demonstrating enhanced cardiac functions in terms of contractile stress and relaxation-contraction rates.^171^ Additionally, the integration of polymers, including AESO plant-extracted polymers, showed excellent mechanical properties and compatibility with traumatized cardiac tissues. This progress signifies a new frontier in cardiac TE through 4D printing, promising personalized treatment for cardiovascular diseases.

**Figure 4:**
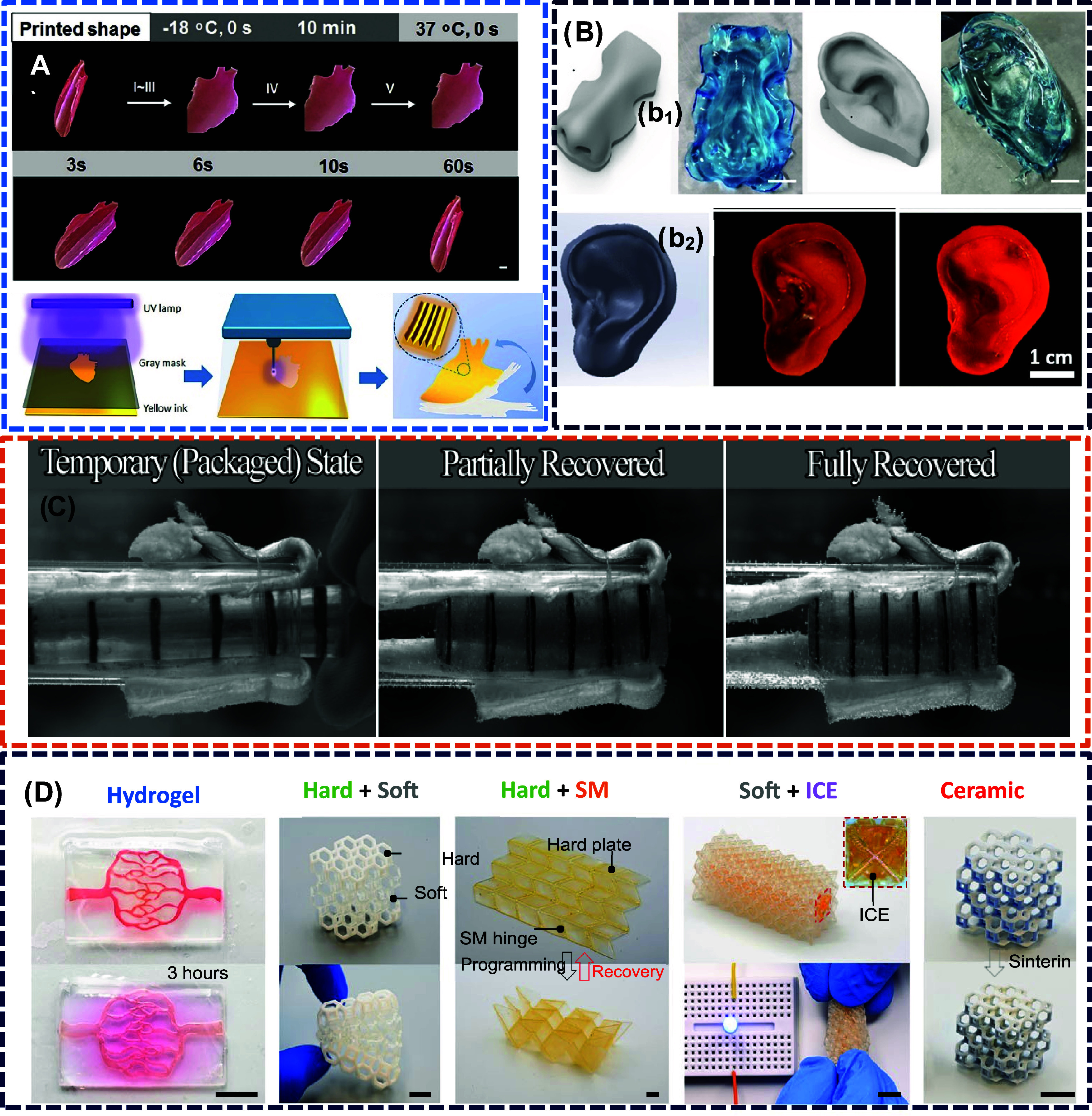
(A) Use of the photolithographic-stereolithographic-tandem printing strategy (PSTS) in soft lithography to outline scaffolds, followed by SLA to imprint micropatterns. This approach enabled the captivating shape transformation of a flat heart construct (adapted with permission from ref 170, copyright 2018 IOP Publishing Ltd.). (B) (b_1_) CAD model and printed of nose and ear auricle with helical fold (adapted from ref 172, under the Creative Commons CC-BY license); (b_2_) the model of ear is printed via DLP with and without addition of CNC (adapted with permission from ref 173, copyright 2019, Springer Nature B.V.). (C) The use of SMP which used in soft tissue repair. The device is inserted into bone, and upon heating at body temperature, it expands (adapted with permission from ref 174, copyright 2008, WILEY-VCH Verlag). (D) The blood vessel which is embedded in transparent hydrogel matrix where blood diffuses with time into matrix. The Kelvin foam structure consists of a soft polymer sandwiched by two hard polymers. The hard polymer is attached to SMP. The ionic conductive octet truss in which IC elastomers are surrounded by nonconductive soft polymer (adapted from ref 175, under a creative commons attribution 4.0 license).

Heart, lung, and trachea were fabricated using a 30% Sil-MA mixture and the DLP technique. When the heart, along with the aorta, the lungs enclosed within the thoracic cavity, and the trachea with its internal lumen, are pushed with the thumb and then released, they return to their previous shape.^104^ The complex helical-shaped ear and nose were also fabricated with hybrid bioink with DLP,^172,173^ as illustrated in Figure 4B­(b_1_ and b_2_), respectively. The pivotal role of 4D printing technology in nerve TE is underscored by its capacity to fabricate microgrooved structures, facilitating the directional growth of nerves.^176^ Utilizing 4D printing, nerve-guiding conduits were intricately constructed through the incorporation of bioink containing acrylated epoxidized soybean oil (AESO), human mesenchymal stem cells (HMSCs), and graphene. The resulting printed structure exhibits bending capability attributable to the AESO-based polymer, while the biomaterial’s conductivity is augmented by graphene-based nanoparticles, thereby promoting the differentiation into nerve cells. The SMP-based AESO recovers its shape at normal body temperature, facilitating the development of smart nerve guidance conduits (NGCs) endowed with multifunctionality for nerve grafting. PEGDA hydrogel is used to make bioink which is powered by cardiomyocyte contraction and the compression modulus of PEGDA hydrogel is compared to human cartilage tissue.^177^

Prior to the addition of gelatin, neural stem cells were distributed throughout a hydrogel based on polyurethane (PU), exhibiting remarkable differentiation and proliferation at room temperature.^178^ The restorative potential of 4D-printed biodegradable hydrogel was demonstrated in the healing of nerve injuries in zebrafish.^179^ Moreover, nerve cells cultured on artificial nerve grafts exhibit remarkable proliferation, adhesion, and differentiation. The innovative application of 4D-printed self-healing hydrogel emerges as a promising strategy for nerve regeneration.^180^ The SMP cylindrical device used to support soft tissue fixation,^174^ as illustrated in Figure 4C, where it is inserted in the bone, and upon heating, it expands and helps the soft tissue to fix. The red blood diffuses into fabricated blood vessels,^175^ as illustrated in Figure 4D. A soft polymer layer is sandwiched between two hard layers to create a Kelvin foam structure. Transformation from a flat to a three-dimensional shape is made possible by a printed Miura-origami sheet made of rigid polymer panels connected by shape memory polymer hinges. Additionally displayed is a flexible ionic conductive octet truss with an IC elastomer core covered in a soft polymer that is not conductive. Finally, to create a pure ceramic architecture, a two-material Kelvin foam structure is fused.

The progress in 4D printing technology signifies a substantial advancement in the domain of soft TE. Researchers may now utilize smart materials and advanced printing processes to produce dynamic, biocompatible structures that replicate the intricate shapes and functional properties of genuine tissues. These advancements have immense potential for various medical uses such as regenerating neural tissue and repairing the heart. It has also been demonstrated how 4D printing can significantly improve patient results.

#### 3.1.2 Hard Tissue Engineering

3D printing is used to create patient-specific implants and scaffolds to replace tissues damaged by defects or injuries. Although these implants are biocompatible, they often lack the ability to support natural regeneration. However, smart materials printed using 3D technology can evolve their characteristics over time. Additionally, these implants can mimic the porous nature of bone, facilitating neural network development and vascularization. Advancements in fabrication through VPP allow for increasingly intricate geometries. The 3D printing in the realm of cartilage TE, the pivotal role of 4D printing takes center stage in the regeneration of damaged cartilage tissue.^181^ This innovative approach leverages 4D printing to fabricate intricately curved cartilage tissue for implantation purposes.^182^ Notably, the application extends to tracheal implants realized through photo-cross-linked silk fibroin-based hydrogel using 4D printing methodologies.^183^ A 3D printer was used to create cell-laden hydrogel structures, subsequently implanted into rabbits for in vivo assessment of their response. Encouragingly, the results demonstrated the promising regeneration of cartilage tissue within the trachea of rabbits.

Further, sol-gel-forming hydrogels were used to repair the bone defect by filling the injured bone segments. The bone was restored by using customized scaffolds that matched the defect and repaired it. The neural network was then integrated to encourage bone development and aid in the healing process,^184^ as illustrated in Figure 5A. Furthermore, the development of artificial fibrocartilage was achieved by precisely controlling during printing, real-time matrix remodeling regulates the collagen fiber orientation,^185^ as illustrated in Figure 5B. A hybrid osteochondral scaffold is developed using various biofabrication techniques to regenerate the cartilage-bone interface. This scaffold features an alginate-gelatin/PCL structure for cartilage and an HAp-loaded PCL scaffold for subchondral tissue. An alginate-gelatin hydrogel composite bioink is used to encapsulate chondrocytes, which are then bioprinted utilizing FDM in conjunction with a PCL framework.^186^

**Figure 5:**
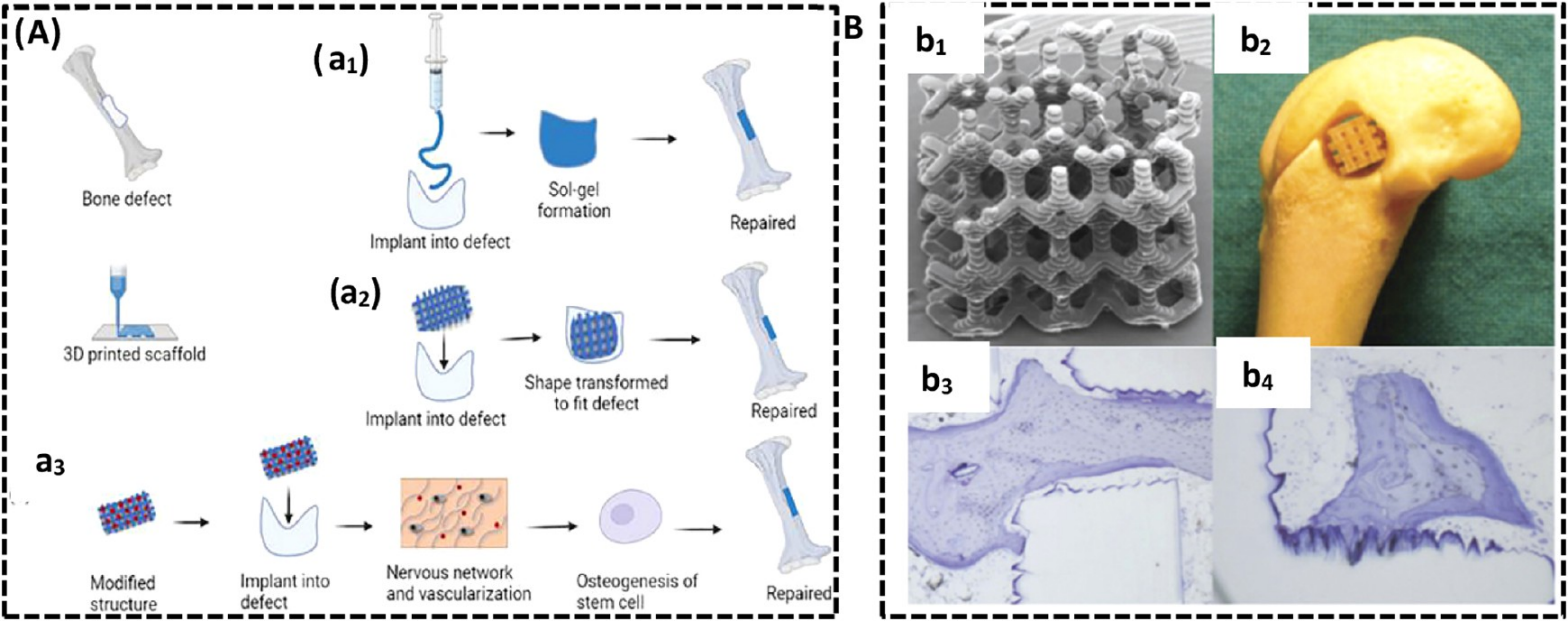
(A) Bone defect recures using biomaterial. (a_1_) A sol-gel-forming hydrogel was injected into the damaged bone, promoting bone repair, (a_2_) a shape-changing scaffold is 3D-printed to match the defect and to facilitate the repair process, (a_3_) 4D printing employed to develop a scaffold that mimics a biological environment, facilitating the formation of a nervous network and encouraging bone growth, thereby improving the bone formation process (adapted from ref 184, under a Creative Commons Attribution 4.0 License). (B) Biocompatible scaffolds using SLA; (b_1_) Utilizing vinyl ester resin to construct the scaffold and capture its structure through SEM imaging, (b_2_) examining the biocompatible scaffolds in their original location, (b_3_) observing newly formed bone with exceptional integration into the host after 8 weeks of implantation, with a detailed zoomed-in perspective, (b_4_) growth and proliferation are enhanced by bone apposition along a surface resembling a sawtooth pattern (adapted with permission from ref 185, copyright 2009, Wiley Periodicals, Inc.).

Additionally, 4D printing facilitates the production of functionally graded polymeric products characterized by tailorable mechanical properties, including the printing of soft muscles around bones using these materials. Such multifaceted applications underscore the versatility of 4D printing in advancing cartilage TE and the creation of complex biological structures.^187^ Further, the tissue scaffolds need to be composed of biocompatible and biodegradable material, and for the growth of the cell, we need scaffold on which it can grow using material extrusion method.^188^

The process of creating biocompatible scaffolds by using SLA involves several sequential stages. Initially, the scaffold is constructed using vinyl ester resin, and its structure is recorded through an SEM imaging technique. Biocompatible scaffolds are then evaluated in their original position. After an 8-week implantation period, newly formed bone exhibiting remarkable integration with the host is observed with a detailed close-up view. Bone growth and proliferation are enhanced along a surface resembling a sawtooth pattern.^185^ Repair of bone fractures and defects was achieved through innovative 4D printing, employing external stimuli for bone grafting.^47,189–191^ This groundbreaking bone grafting technique harnesses the capabilities of highly intriguing 4D printing technology.^192^ Specifically, scaffolds with photothermal responsiveness were meticulously crafted by combining β-tricalcium phosphate (TCP) and PLA-TMC, enriched with osteogenic peptide and BPNSs.^193^ The application of near-infrared radiation triggered a restructuring of the scaffolds, as evidenced by in vivo assessments demonstrating a significant improvement in bone regeneration in rat cranial defects. Importantly, the mechanical attributes of these scaffolds closely mimicked those of the initial trabecular bone, particularly under physiological temperatures.

Furthermore, SMP-based scaffolds were compressed into temporary shapes and reverted to their original form at a specific physiological temperature. These SMP-based structures were 3D-printed and implanted for mandibular bone repair, utilizing a HAp/PCL-based SMP.^194^ These breakthroughs demonstrated the profound capacity of 4D printing to produce highly efficient, biocompatible implants and scaffolds. Through ongoing improvements in these technologies, the discipline is progressing toward the achievement of seamlessly integrating artificial structures with natural tissue for complete regeneration.

### 3.2 Drug Delivery Devices

Drugs have long been instrumental in improving human health, with their chemical compounds rigorously tested and administered to achieve therapeutic effects. Over time, the methods of drug delivery have evolved, becoming increasingly complex and innovative in the quest for more effective treatments for specific diseases. The emergence of 4D printing has heralded a new era in drug delivery, offering unprecedented control over medication release. A biodegradable, shape-memory intestinal drug delivery device is fabricated via 4D printing using a photopolymerizable resin composed of poly­(β-aminoester) (PBAE) and stearyl acrylate (C18-acrylate).^195^ The device is designed to undergo programmed compression into a temporary shape for encapsulation within an enteric-coated oral dosage form, followed by thermally triggered expansion upon reaching intestinal conditions (37 °C, pH 6.8). This approach leverages the shape-memory effect to enable self-expansion in the small intestine, as illustrated in Figure 6A, thereby enhancing drug permeation through localized mechanical interaction with the intestinal mucosa while minimizing gastric exposure. The material exhibits rapid biodegradation (within 6–12 h) under physiological conditions, mitigating risks of intestinal obstruction, while maintaining high elasticity (>700% strain) and mechanical strength (>7.5 MPa) for reliable deployment. By incorporating a natural photoabsorber (lawsone) and optimizing resin composition, the system demonstrates improved cytocompatibility and tunable thermomechanical properties. This addresses key challenges in oral macromolecular delivery, including poor bioavailability and gastric degradation, by combining precise spatial control with stimuli-responsive material behavior, offering a promising strategy for gastrointestinal-targeted drug delivery.

**Figure 6:**
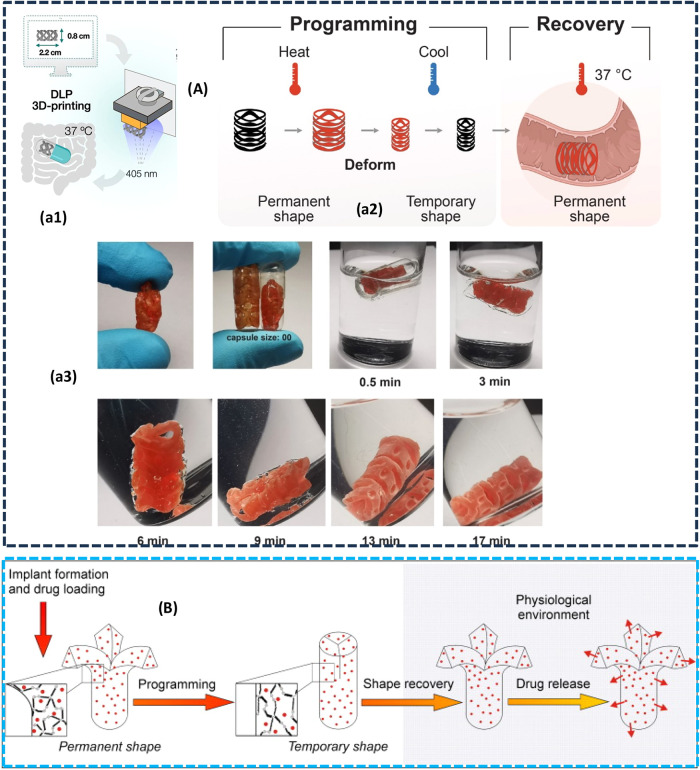
(A) (a1) Drug delivery in intestine, (a2) programmed and recovery shape of drug delivery device, (a3) programmed and permanent shapes of the delivery device were compared after incubation in water at 37 °C, with the device’s reddish coloration resulting from incorporated lawsone (adapted with permission from ref 195, Copyright 2009, Elsevier B.V.) (B) Controlling the shape recovery of a polyester urethane SMP network within physiological conditions and temperatures, while also regulating the release of drugs (adapted with permission from ref 196, copyright 2009, Elsevier B.V.).

SMEs are also utilized for controlled drug delivery where a drug is released by manipulating the configuration of elastomers, causing them to open and release the drug,^196^ as illustrated in Figure 6B. Drug release can be controlled by adjusting concentrations,^197^ using various geometries,^198^ and improving drug solubility in water.^199^ One promising avenue lies in the application of magneto-active polymers (MAPs), which possess the unique ability to induce localized heating, which is particularly valuable in cancer therapy. Acknowledging the challenges inherent in chemotherapy, such as drug resistance and significant patient toxicity, the potential of MAPs for targeted drug delivery is widely recognized. Furthermore, these nanomaterials hold promise for hyperthermia treatment, presenting a multifaceted approach to combating disease. To achieve this, magneto-active NPs can be utilized to release energy when subjected to high-frequency magnetic fields. This mechanism enabled the eradication of malignant cells or the targeted release of chemotherapeutic agents precisely at the tumor site.^200–205^

A water-soluble photoinitiator was used to covalently link hydrogels with polymers in a new DLP-based multimaterial 3D printing technique, creating hybrid structures with high water content and stretchability.^206^ Ionic conductors, strain sensors with antidehydration layers, spatially graded 3D-printed menisci, 4D-printed drug-delivering stents, reinforced hydrogel composites, and single-network two-way shape memory resins for soft robotics and medical devices are a few examples of applications.^177^ The resin consisting of two PU-based oligomers with different transit temperatures will be used for the fabrication of two-way shape polymers via a DLP printer. The switching segments of PCLDA and PPDLDA demonstrated notable mean shape fixity ratios exceeding 97% and high shape recovery ratios exceeding 89%. Leveraging these properties, the material was employed in the fabrication of biodegradable soft actuators capable of effectively gripping and releasing objects.

Soft, biodegradable microswimmers, fabricated through 2PP using GelMA material, demonstrate complete degradability. Throughout the culture period, cell-secreted enzymes contribute to their gradual breakdown, concurrently fostering cell attachment and proliferation. Furthermore, a magnetically propelled double-helical microswimmer, responsive to external stimuli such as light, exhibits controlled release of the chemotherapeutic drug doxorubicin. These light-triggered drug release mechanisms are incorporated into magnetically actuated, biocompatible, and biodegradable chitosan-based microswimmers, all of which are precisely engineered through the 2PP.^204^

Drug delivery systems mostly employ stimuli-responsive 4D printing to precisely regulate drug release.^207^ Treating the gastrointestinal tract, this system utilizes a PNIPAM-AAc/poly­(propylene fumarate) material to achieve thermoresponsive drug release.^208^ The designed capsule features a core–shell hydrogel system, with the core comprising ethylene glycol, poly­(vinyl alcohol), and biomolecules. The shell incorporates gold nanorods (AuNRs) and poly­(lactic-*co*-glycolic acid) (PLGA). External laser stimuli induce AuNRs rupture, facilitating drug release. Additionally, shape-morphing microrobotics, exemplified by the shape-morphing micro fish (SMMF), are engineered to deliver Doxorubicin (DOX). The SMMF exhibits controlled opening and closing of its mouth under phosphate-buffered saline and slightly acidic conditions, respectively. To assess effectiveness, an artificial vascular network is established, demonstrating the SMMF’s capability to release drugs for treating cervical cancer cells.^209^ The utilization of advanced materials and 4D printing technology in drug delivery demonstrates the potential to improve therapeutic outcomes. These systems enable precise control over drug release and utilize stimuli-responsive processes, which can lead to substantial breakthroughs in targeted and successful treatments.

### 3.3 Biomedical Devices

Biomedical engineering has been revolutionized by 4D printing, which creates minimally invasive biomedical devices. These devices include cardiovascular stents, microfluidic devices, soft grippers, actuators, disease detectors, and wound healers.^210^ Cardiovascular stents are capable of changing their shapes in response to specific stimuli and, in some cases, releasing drugs upon stimulation.^206^ Other devices are also fabricated through VPP printing, showing considerable promise for treatment and various applications.

#### 3.3.1 Micromachines

Micromachines are used in biomedical engineering to control fluids and act as grippers during minimally invasive medical procedures. Robotic surgical tools can be used to manipulate tissue and aid in surgical operations. These tools can grab and manipulate tissue within the body without the need for big incisions. They give means for carrying out accurate and complicated therapies with the least amount of danger or injury to the patient.

The fabrication of micromachines for biomedical applications is evolving. In this investigation, the incorporation of FeNi nanoparticles (NPs) into a photopolymer matrix was undertaken to leverage their favorable characteristics, as illustrated in Figure 7A, such as soft magnetic responses and high magnetizations.^211^ The utilization of field-assisted printing was employed to induce magnetic anisotropy by organizing laser-synthesized FeNi particles into uniaxial magnetic strands measuring up to 500 μm in length. The advantageous attributes of the FeNi, including their diminutive size and uniform dispersion, allowed for the achievement of magnetic responsivity with a minimal addition of only 0.02 wt %. This approach not only mitigates the impact on the mechanical properties of the composite but also affords enhanced control over the resultant magnetic properties. The strategic use of FeNi in the photopolymer matrix, coupled with field-assisted printing, demonstrates a promising avenue for tailoring magnetic composites with a minimal impact on mechanical integrity, thereby expanding the scope of applications in various fields.

**Figure 7:**
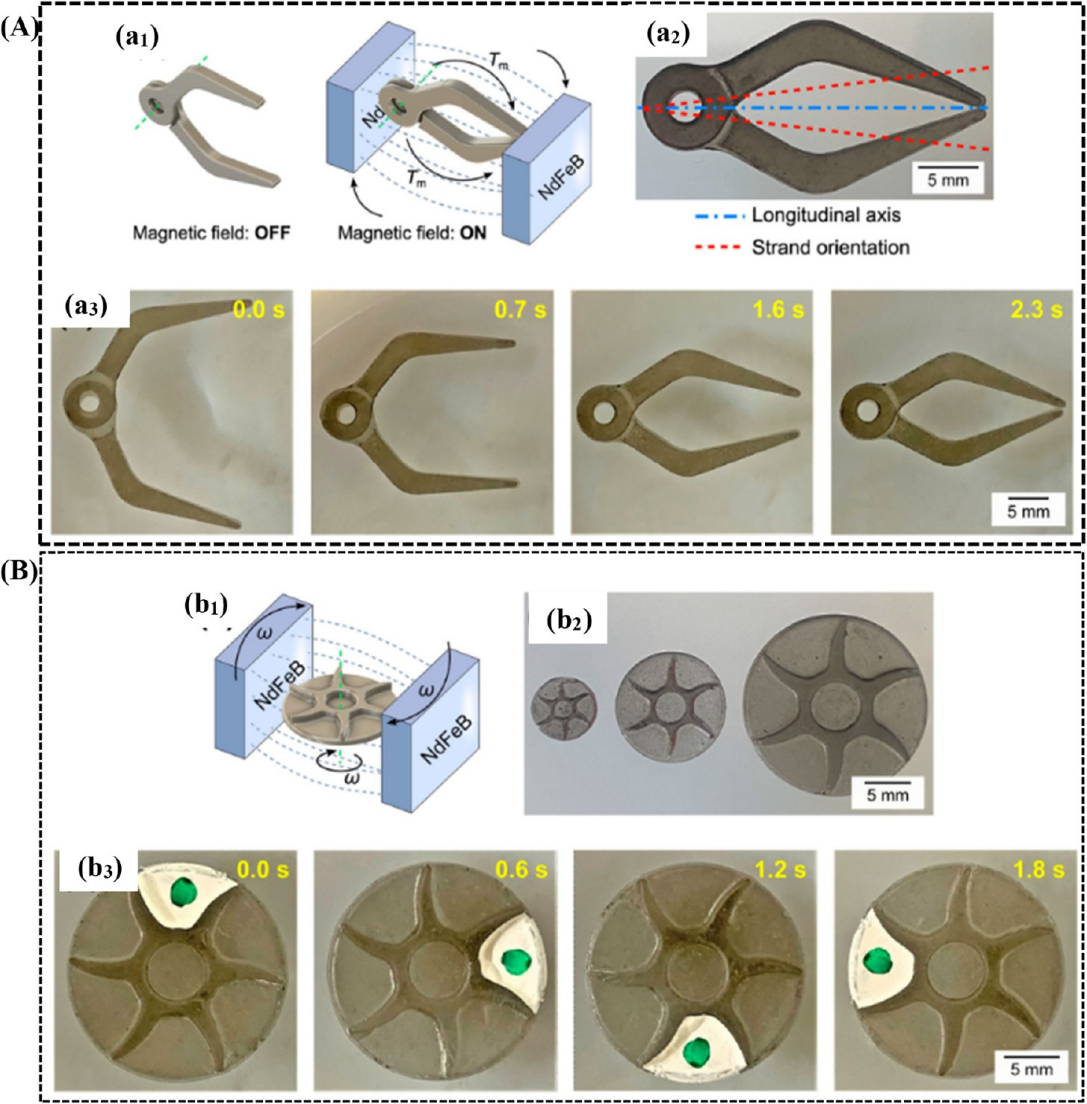
(A) Magnetically responsive gripper: (a_1_) The contraction and expansion of gripper which is based on magnetic field, (a_2_) image of gripper with marked orientation of strands, (a_3_) gripping actuation time sequence which was placed on water. (B) Magnetically responsive impeller: (b_1_) The impeller induced by the magnetic field, (b_2_) impellers differentiate with diameters 7, 12, and 20 mm, (b_3_) rotating impeller time sequences which was placed on water (adapted with permission from ref 211, copyright 2023, Taylor & Francis).

The MRP impeller is also used in sealed engines for microfluidic applications and in controlled flow for remote and wireless applications.^212^ The investigation effectively highlighted the creation and activation of MRP gripper and impeller actuators utilizing a mere 0.02 wt % of FeNi NPs,^211^ as illustrated in Figure 7B. Notably, the MRP gripper, incorporating only 0.04 wt % FeNi NPs, demonstrated actuation times of under 3.0s, underscoring its viability for remote robotic tasks. The magnetic anisotropy inherent in the MRPs enhances their appeal for wireless microfluidic applications, as illustrated by monolithic MRP impellers exhibiting rotation frequencies surpassing 1 Hz when immersed in water.^211–213^

A conductive hydrogel, distinguished by its high conductivity and biodegradability, was synthesized using a projection microstereolithography in alkaline solutions. Additionally, this material exhibits stretchability and is responsive to external stimuli, including variations in temperature, pH, and chemical composition, influencing both its conductivity and degradation characteristics. These hydrogels find application in wearable devices, effectively measuring body movements and detecting signals from the fingers, knees, and vocalizations. These breakthroughs demonstrate the potential of combining modern materials and creative printing techniques to create complex biomedical devices that increase patient care and improve the accuracy of medical operations.

#### 3.3.2 Soft Robotics

The field of soft robotics in biomedicine is being revolutionized by 4D printing, as SMP are being used in heart stents, grippers for holding objects, hydrogel-based locomotive soft robots, and microactuators.^214^

The micromechanical soft actuators were fabricated through the application of 2PP,^215^ as illustrated in Figure 8A, leveraging acoustic signals as a potent energy source. A key feature of these actuators involves the encapsulation of air bubbles within the capsules, a process achieved through the utilization of 3D printing technology. The selected material for constructing these actuators is TPETA, a soft, biocompatible biopolymer. This choice of material is driven by its pliable nature and its compatibility with biological systems. The orchestrated integration of 2PP, 3D printing, and TPETA material manifests in the creation of soft actuators, demonstrating promising attributes for applications within the realm of biotechnology and allied disciplines.

**Figure 8:**
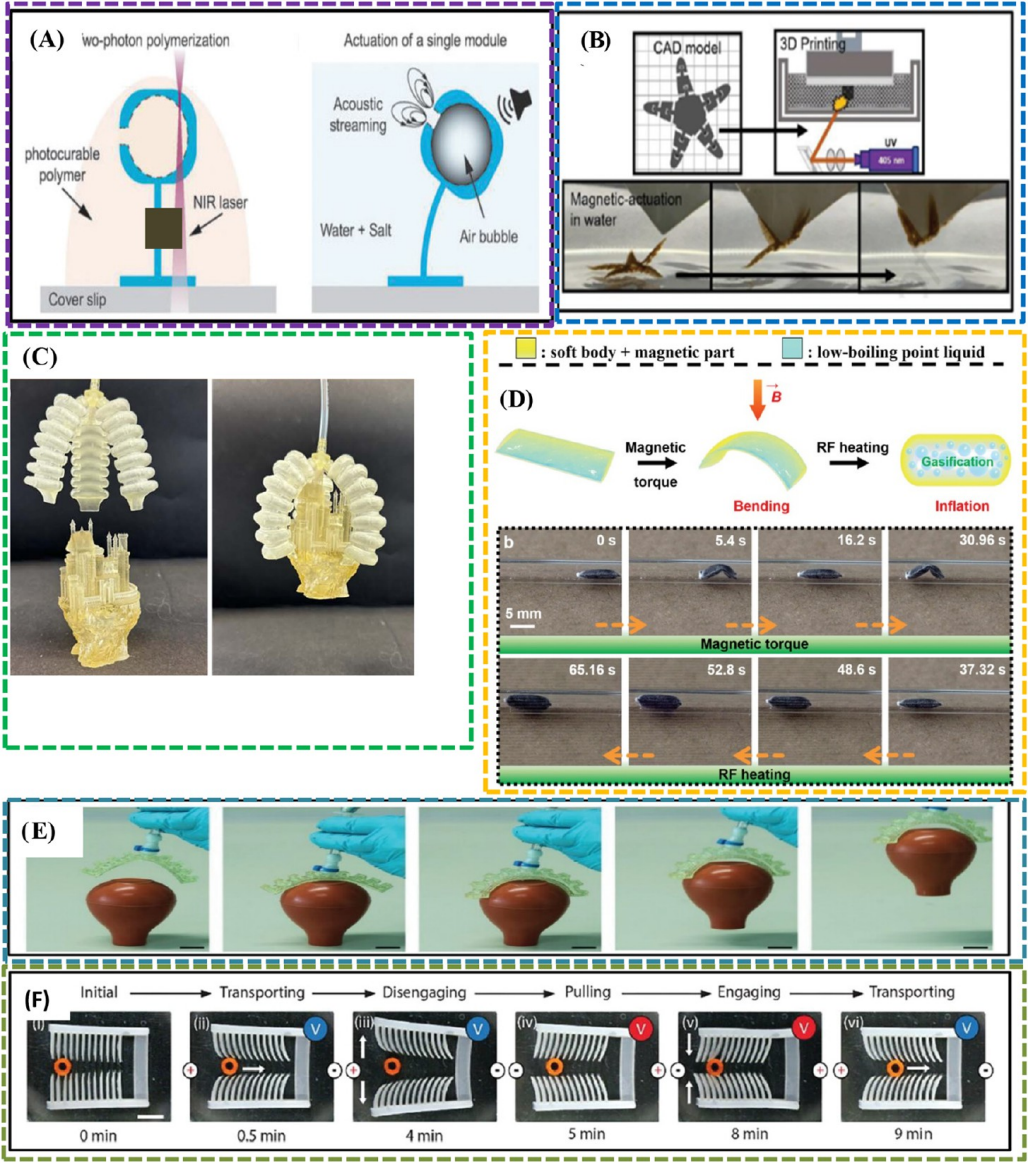
(A) 3D-printed acoustically programmable soft microactuators using 2PP DLW technique (adapted with permission from ref 215, copyright 2023, Mary Ann Liebert, Inc.). (B) 3*D*/4D-printed BSSA starfish-shaped superparamagnetic stimuli-responsive hydrogel fabricated by SLA (adapted from ref 153, under a creative commons attribution license). (C) Tri-y gripper parts and assembly (adapted from ref 154, under a Creative Commons Attribution License). (D) Schematic diagram depicts the dual-mode actuation of the soft sheet-like device, highlighting bending induced by magnetic torques and expansion triggered by combined magnetic and radio frequency (RF) heating. (b) Sequential images capture the locomotion and volumetric expansion of the phase-change magnetic soft robot as it navigates within a confined glass tube, demonstrating its response to externally applied magnetic torques and RF-induced heating (adapted with permission from ref 216, under creative common CC BY license). (E) DLP 3D-printed pneumatic soft actuator with two fingers capturing a soft rubber pipet bulb (adapted with permission from ref 217, copyright 2022, Wiley-VCH GmbH). (F) 3D-printed electroactive hydrogel-based locomotive soft robot (adapted with permission from ref 218, copyright 2018, American Chemical Society).

In this research endeavor, a meticulously optimized methodology has been employed for the integration of superparamagnetic iron oxide NPs, each possessing a diameter of 10 nm, into a photoresin formulated with constituents comprising water, acrylamide, and PEGDA. The focus of the approach lies in the achievement of an infusion of up to 2 wt % of these nanoparticles, ensuring not only enhanced homogeneity but also the mitigation of agglomeration phenomena during the printing process. This meticulous optimization is aimed at advancing the state-of-the-art in nanoparticle incorporation methodologies within polymeric matrices, particularly in the realm of photoresins, thereby contributing to the refinement of printing techniques for diverse applications. The printed starfish-shaped hydrogel demonstrated, as illustrated in Figure 8B, robust mechanical stability with an elastic modulus of 1.8 MPa and limited shape deformation (10% expansion).^153^ Activation of magnetic properties in each limb through a remote magnetic field renders these hydrogels suitable for integration into degradable soft robotics, offering controlled responses to external stimuli. Multilateral actuators and microactuators use biodegradable materials.^219^ Multimaterial actuators surpass their single-material counterparts in both assembly simplicity and superior deformation controllability.

Furthermore, the tri-y gripper parts and assembly are shown, including the gripper holding a ball, and small and large irregular shapes that were 3D-printed,^154^ as illustrated in Figure 8C. The progress in soft robotics highlights the significant impact that 4D printing can have on the field of biomedicine. Researchers are using advanced materials and printing processes to develop medical devices with improved usefulness, adaptability, and biocompatibility. The dual-mode actuator walks toward the target point driven by magnetic torque and subsequently inflates due to the combined effects of magnetic fields and radio frequency heating.^216^ A low-boiling-point liquid embedded in the material vaporizes upon RF heating, leading to the observed inflation behavior, as illustrated in Figure 8D.

Soft biohybrid robots, composed of both soft materials and cells, have demonstrated the capability to detect, respond to, and adapt to environmental stimuli in real-time. These biohybrids exhibit lifelike functionalities such as swimming, walking, and pumping.^220^ Soft robotic systems with cell integration provide new possibilities for creating robots that can dynamically detect and react to intricate environmental stimuli.^221^ A biological machine is designed to walk and use a multimaterial SLA printer. The soft material known as PEGgel, distinguished by its inherent self-healing properties, serves as a pivotal component in the development of self-healing pneumatic actuators tailored for applications in the realm of soft actuators and robotics,^217^ as illustrated in Figure 8E. Employing DLP technology, self-folding origami structures have been successfully engineered with water serving as an external stimulus. The fabrication involves the creation of a biodegradable sheet capable of transitioning between two distinctive surfaces, namely, a saddle and a dome.^222^

The electroactive hydrogel, produced through DLP-based microprinting techniques, is employed in soft robotics and locomotion applications. It holds significant potential for utilization in artificial muscles,^218^ as illustrated in Figure 8F. Such developments make 4D-printed actuators well suited for future biomedical technologies that require adaptable, minimally invasive, and personalized solutions.

#### 3.3.3 Biosensors and Medical Devices

Biosensors are making considerable progress in disease detection, wound healing, and supporting the body in performing its tasks. 4D printing plays a key role in advancing biosensor-based biomedical devices by incorporating smart materials for the detection and treatment of diseases.^223^ Wound management aims to enhance the protection and healing of wounds, as illustrated in Figure 9A,B. To achieve this, advanced strategies are being employed. One such approach involves the use of a multifunctional patch, which is integrated with specialized dressing materials. This integration helps prevent irritation and chemical reactions on the skin. An example of such a patch is GelDerm, which has been specifically designed to detect bacterial infections and release antibiotics. The detection process involves measuring the pH changes using a calorimeter, which serves as an indicator for the presence of bacteria.^224^ The sensor uses the activation of strain as external stimuli using a material biopolymer PEGDA for application in soft electronics, strain sensors, and cardiovascular stents.^206^ A DLP-based printing technique is used to fabricate the stimuli-responsive analytical device for the determination of the urea and glucose,^225^ as illustrated in Figure 9C. The study introduces a novel 4D-printed needle panel meter for enzymatic detection of urea and glucose, fabricated through digital light processing (DLP) 3D printing using a dual-resin system. The device consists of a nonresponsive base layer made from *tert*-butyl acrylate, 1,6-hexanediol diacrylate, and a photoinitiator, combined with a pH-responsive layer incorporating 2-carboxyethyl acrylate (CEA, 25% v/v) that swells at pH > p*K*_a_ (∼4.6–5.0) due to electrostatic repulsion of dissociated carboxyl groups. The sensor demonstrates excellent performance, with detection limits of 4.9 μM for urea and 7.0 μM for glucose across a 0.1–10 mM linear range, validated in biological samples (urine, serum, and plasma) with 96–107% recoveries. Its reusable design, simple visual/caliper-based readout, and low production cost highlight its potential as a practical point-of-care diagnostic tool, advancing 4D-printed stimuli-responsive biosensors for decentralized healthcare applications. These advancements highlight the capacity of biosensors to enhance biological applications and patient outcomes. With smart materials and innovative 4D printing methods, these devices can completely transform the biomedical engineering area.

**Figure 9:**
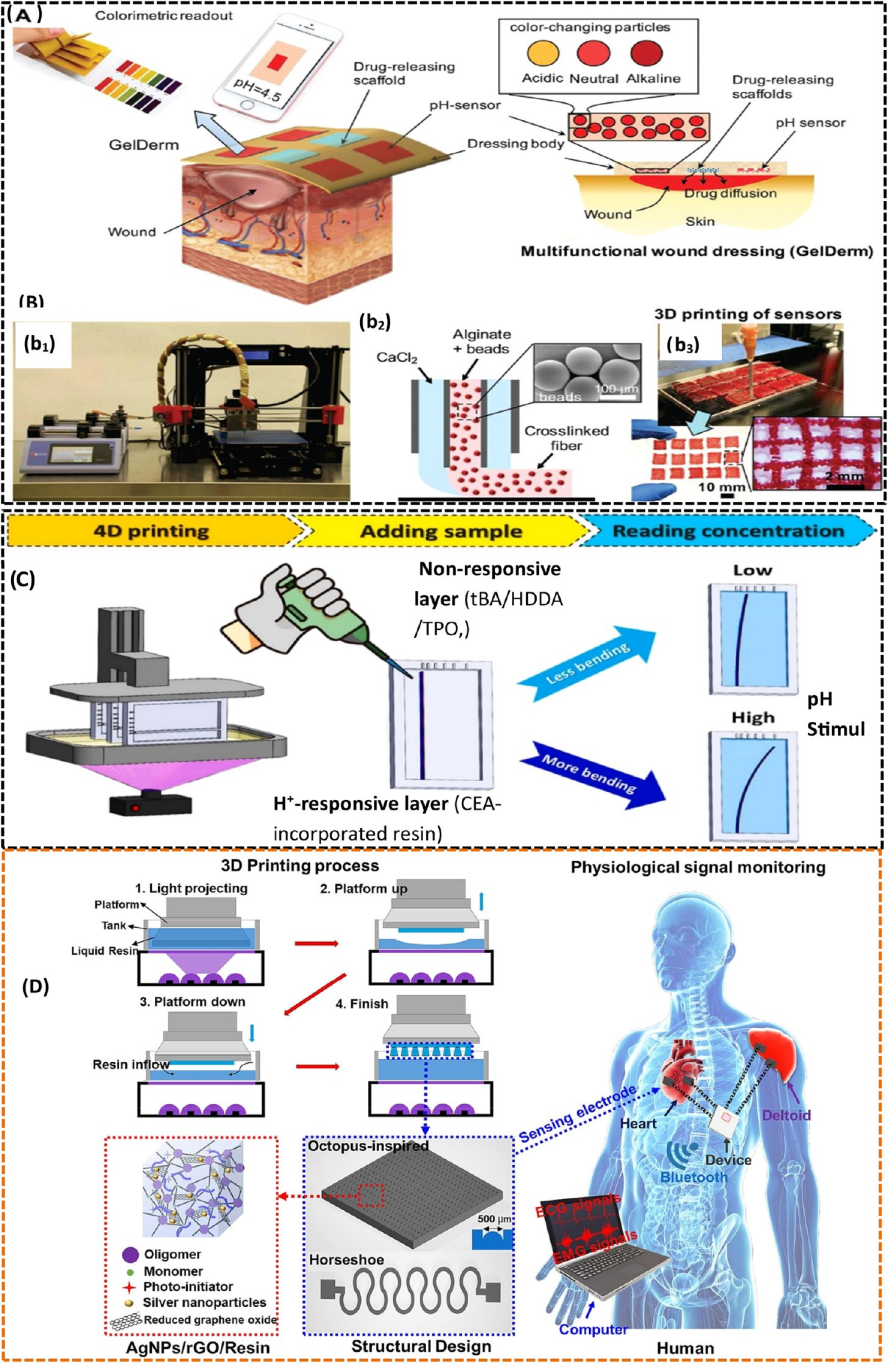
(A) Schematic diagram of the multifunctional wound dressing (GelDerm) for bacteria detection and releasing of antibiotics (adapted with permission from ref 224, Copyright 2017, John Wiley and Sons). (B) (b_1_) The printer for the fabrication of biomaterial; (b_2_) the materials; (b_3_) the fabrication of the pH sensor for detection of bacterial infection (adapted with permission from ref 224, Copyright 2017, John Wiley and Sons). (C) Fabrication of an analytical device to determine the urea and glucose (adapted with permission from ref 225, copyright 2023, Elsevier B.V.). (D) Highly conductive resins used to print wearable smart electronic devices for monitoring of physiological signals (adapted with permission from ref 226, copyright 2024, Elsevier B.V.).

Real-time health monitoring and personalized smart devices are revolutionizing the health sector, thanks to emerging technologies such as the Internet of Things, Big Data, and the 5G network. 3D-printed, wearable, flexible sensors can help to treat and prevent chronic diseases by monitoring physiological signals, such as electroencephalograms (EEG), electrocardiograms (ECG), and electromyograms (EMG).^227^ VPP is a suitable printing technology for developing flexible resins by modifying them with flexible functional groups, such as urethane. Furthermore, the introduction of conductive particles, such as graphene and carbon black, induces conductive properties that enable the printing of flexible sensing electrodes for physiological monitoring,^226^ as depicted in Figure 9D.

The DLP 3D printing technique has great potential to fabricate customized, bioresorbable airway stents with elastomeric properties, as illustrated in Figure 10A. Conventional silicone stents, while effective, are prone to migration and require invasive removal, posing risks to patients. To address this, researchers develop dual-polymer photoinks composed of methacrylated poly­(d,l-lactide-*co*-ε-caprolactone) copolymers, which enable high-resolution printing of stents with tunable mechanical properties and controlled degradation.^228^ The optimized stents exhibit elasticity comparable to that of commercial silicone stents and maintain structural integrity under physiological conditions. In vivo studies in rabbits reveal that the stents remain stable for 7 weeks before gradual resorption, with histological analysis confirming biocompatibility and reversible tissue responses. This work represents a significant advancement in personalized medical devices, combining patient-specific design with biodegradability to improve outcomes in airway obstruction therapy.

**Figure 10:**
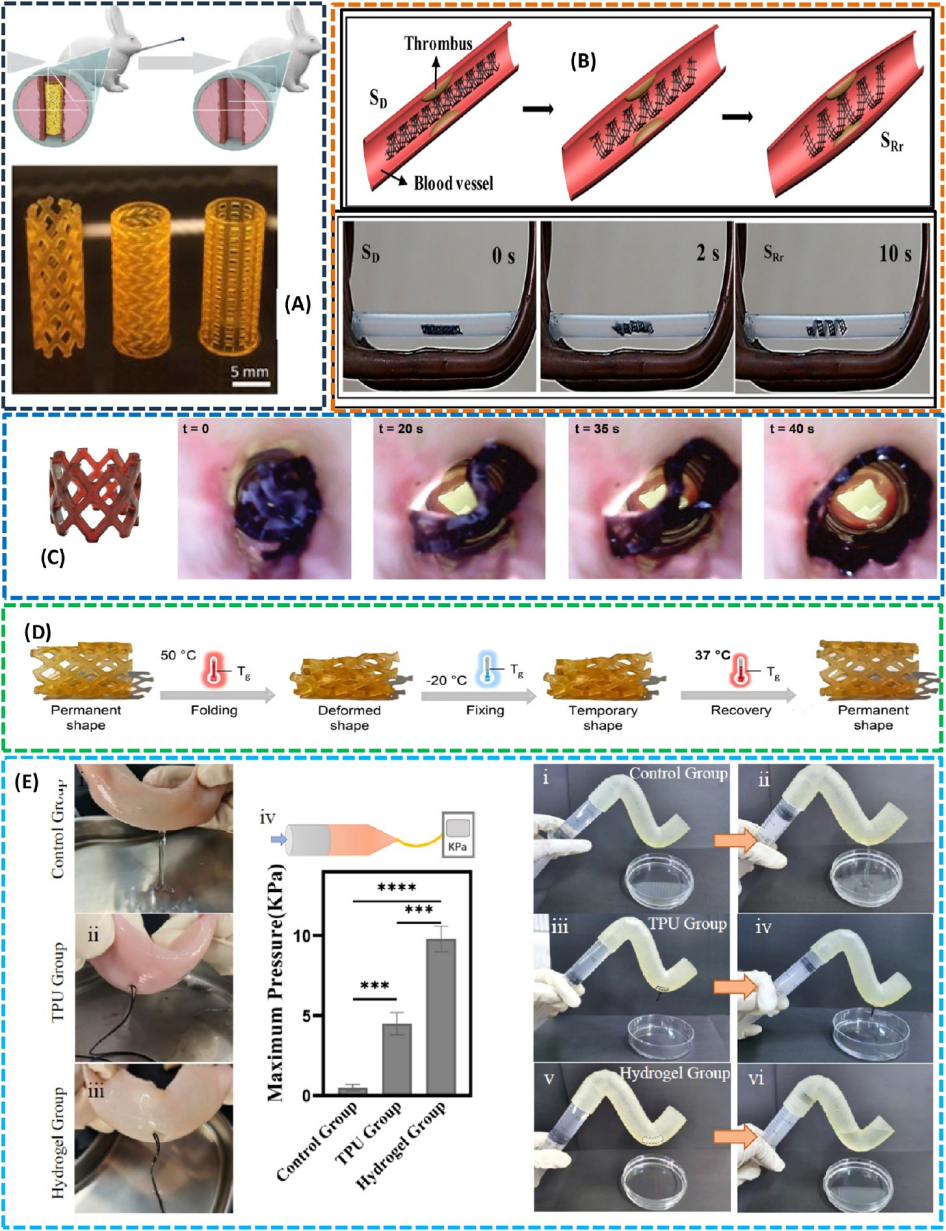
(A) Rabbit CT scanned geometry extracted for stent fabrication. Different designs of the airway stent were fabricated using DLP (Reproduced from ref 228, under a creative commons attribution license 4.0­(CC BY)). (B) Intervascular stent for curating thrombus through 4D fabrication (Adapted with permission from ref 229, copyright 2017, American Chemical Society). (C) Composite-based 3D-printed stent with human-scale dimensions (height: 12 mm, diameter: 16 mm, wall thickness: 1.6 mm).  Following shape programming, the stent demonstrates near-infrared (NIR) light-triggered shape recovery within an ex vivo porcine intestinal segment. (Adapted with permission from ref 230, without Copyright 2022 Advance Science). (D) 4D-printed drug-loaded stents are programmed by folding above *T*_g_ and cooling below *T*_g_ to fix their temporary shape. Upon delivery, they self-expand and gradually degrade while releasing drugs. (Adapted with permission from ref 231, creative commons CC-BY license. Without Copyright 2023, Elsevier). (E) Porcine intestine with 2 mm hole: (i) Unplugged control, (ii) 3D-printed TPU stent, (iii) 4D-printed bilayer hydrogel, (iv) maximum intraluminal pressure tolerance (*p* < 0.001, *p* < 0.0001, one-way ANOVA). Resin intestinal model with 2 mm hole: (i–ii) unplugged control, (iii–iv) TPU stent, (v–vi) bilayer hydrogel. (Adapted with permission from ref 232, copyright 2022, Elsevier).

In some cases, stents were developed using shape memory polymers and nanocomposites, allowing control through both materials’ properties. The remote actuation system proved to be an effective method for creating 4D active vascular stents capable of changing shape and offering multifunctional properties,^229^ as illustrated in Figure 10B. The development of microstents is significantly influenced by the consideration of biodegradability, which is paramount in shaping their mechanical characteristics.^233^ SMP-based stents were developed by the researchers, demonstrating a stimulus response to thermal changes, and were fabricated through 4D printing for the purpose of blood vessel expansion. The 4D-printed stents were crafted to be responsive to both thermal and pH stimuli, with a strong correlation being observed between pH and material stiffness.^234^

The composite-based 3D-printed stent, as illustrated in Figure 10C, with dimensions of H 12 mm, Ø 16 mm, and a thickness of 1.6 mm, was fabricated using a digital light processing (DLP) printer and a multifunctional biomedical ink composed of a poly­(DLLA-*co*-CL) methacrylate copolymer and AuNRs functionalized with a thiolated polymeric ligand. The stent was designed to exhibit shape-memory properties, enabling it to undergo programmed deformation and subsequent near-infrared (NIR) light-triggered shape recovery.^230^ The material’s transition temperature, tuned to near physiological conditions, allowed for facile deployment and expansion upon irradiation with an 808 nm laser. The ex vivo demonstration in a porcine intestinal segment confirmed the stent’s ability to rapidly recover its original shape within 40 s of NIR exposure, effectively reopening the obstructed lumen.

Further, 4D-printed biodegradable stents were fabricated using poly­(DLLA-*co*-TMC) methacrylates via heat-assisted DLP.^231^ These shape-memory stents incorporated 1 wt % drugs (levofloxacin/nintedanib) and exhibited tailored transition temperatures (25–42 °C). Programmed by heating above the glass transition temperature (*T*_g_) and freezing below *T*_g_, they fully recovered at 37 °C within 3.5 min, as illustrated in Figure 10D. The stents demonstrated sustained drug release (levofloxacin: burst; nintedanib: gradual) while maintaining cytocompatibility and degrading into hydrogels for over 10 weeks, offering potential for personalized, multifunctional medical implants.

In a recent study, Paunovic et al.^232^ evaluated the plugging efficacy of a 4D-printed bilayer hydrogel composed of acrylamide-acrylic acid/cellulose nanocrystal (AAm-AAc/CNC) with Fe^3+^ coordination in porcine intestines and a resin intestinal model, each with a 2 mm hole to simulate enteroatmospheric fistulas (EAFs). The fabricated bilayer hydrogel was compared against a 3D-printed thermoplastic polyurethane (TPU) stent and a control group (no plugging), as illustrated in Figure 10E. Results demonstrated that the 4D-printed hydrogel achieved complete sealing with no leakage, sustaining pressures up to ∼9.8 kPa, significantly higher than the TPU group (∼4.5 kPa) and control (∼0.5 kPa) (*p < 0.0001, one-way ANOVA). In the resin model, the hydrogel conformed seamlessly to the curved defect, whereas the rigid TPU stent failed to adapt, leading to persistent leakage. These findings highlight the superior adhesion, flexibility, and dynamic shape-morphing capability of the 4D-printed hydrogel, making it a promising solution for minimally invasive EAF closure.

## 4 Current Challenges and Future Perspectives

VPP holds a significant advantage concerning resolution, surface quality, and design flexibility over other additive manufacturing methods. However, restricted material options, confined to photopolymer resins, underscore a critical necessity for the innovation of a diverse range of cost-effective materials with outstanding properties compared to those of existing counterparts. While computational models have demonstrated their utility in anticipating the performance of linear and rigid materials using data-driven approaches, the prediction of stimuli-responsive materials remains challenging due to their nonlinear locomotion characteristics.^235^ In the near future, the outcomes of these prognostications, coupled with the accrual of empirical data, are anticipated to build a substantial database. This repository can then be harnessed by artificial intelligence systems for the exploration and advancement of innovative smart materials.

The biomedical field has already experienced the influence of 4D printing despite being a nascent technology. Envisaging rapid and consistent expansions, the realization of 4D printing’s full potential is anticipated gradually. This realization hinges on the discovery of novel biocompatible dynamic materials and the development of cost-effective 3D printers with high resolution. Further, bioabsorbable materials represent a burgeoning field of investigation, given their numerous clinical applications spanning a wide range of disciplines. These applications encompass bioabsorbable stents^236^ and vascular scaffolds, drug delivery systems, gastroenterology, as well as dental and periodontal devices.^236–241^

Potentials of 3D printing techniques such as VPP are significant for crafting intricate dynamic structures, intelligent medical devices, and even complex human organs.^242^ Despite its promising applications, the concept of 4D bioprinting is still in its initial stages and its practical implementation in clinical settings remains constrained. The challenge is heightened by the formidable task of predicting the deformation in 4D printing, given the current lack of comprehensive computational modeling. Moreover, the development of bioink materials tailored for 4D printing necessitates careful consideration of both biocompatibility and stiffness. Additionally, the exploration of multiple-responsive stimuli is crucial in advancing 4D bioprinting, acknowledging that in vivo environments often involve more than one stimulus. Furthermore, dedicated efforts should be directed toward creating biopolymer-based products for the biomedical sector through 4D printing. This is particularly relevant in scenarios where the 4D-printed components need to remain unresponsive to specific stimuli, such as variations in temperature and pH.

In biomedical research, VPP technology is pivotal for fabricating bone grafts and bioactive scaffolds.^243,244^ Stereochemistry demonstrates significant improvement in mechanical properties and degradability with the same compound ratio for scaffolds.^243^ The choice of materials such as HAp, biphasic calcium phosphate (BCP), β-tricalcium phosphate (β-TCP), and polypropylene fumarate (PPF)^245^ requires careful consideration. HAp stands out for bone grafts due to its excellent mechanical and bioactive properties.^246^ BCP and β-TCP, while lacking mechanical strength compared to HAp, offer strong bioactivity, printability, and functionality.^247,248^ Likewise, PPF, with commendable mechanical properties and cytotoxic response, lacks bioactivity.^249^ The challenge lies in synthesizing these materials using VPP technology, with the aim of a synergistic blend to overcome individual limitations. This interdisciplinary approach drives progress in advanced biomaterials, pushing the boundaries of regenerative medicine and enhancing solutions for bone TE.

In dentistry, the selection of an appropriate biomaterial using the VPP technique is of paramount importance. While ceramic-metal alloys stand as the gold standard in dental applications, the biomaterials zirconia and lithium disilicate, despite demonstrating commendable mechanical properties, present certain limitations that necessitate careful consideration in the context of VPP technology. Notably, zirconia exhibits a drawback concerning its incompatibility with UV light, posing a constraint on its effective utilization in VPP processes.^250^ On the other hand, lithium disilicate emerges as a more suitable alternative; however, both materials lack essential bioactive properties. This underscores the need for further exploration and innovation in biomaterials compatible with VPP techniques that can seamlessly integrate strong mechanical characteristics with bioactive functionality. It is imperative to address these limitations, particularly in the case of PMMA, which is deemed unsuitable due to its propensity for cracks and fractures. This research aims to advance the understanding and development of biomaterials tailored for VPP applications in dentistry, ensuring optimal performance, durability, and biocompatibility for improved clinical outcomes.^251^

In TE, PCL-based materials with self-healing properties are employed to construct small intestine scaffolds, while PTMC finds application in TE scaffolds and meniscus implants.^252–254^ VPP technology for fabricating self-healing tissues or organs is an untapped frontier.^255,256^ Despite the advancements in 3D printing, the use of VPP techniques to 3D print biocompatible materials, including feldspathic ceramic,^257^ PLLA,^258^ PEG-DMAP,^259^ chitosan bioink,^260^ PTHF-DA,^261^ TMPTMA, nanocrystalline cellulose,^262^ nanofibrous silk fibroin,^263^ and z-gel^263^ remains underexplored. These materials exhibit promising biocompatibility, yet their integration into VPP for medical applications is an ongoing challenge. The evolution from patient-specific to mechanically fabricated medical parts with enhanced bioactivity and functionality reflects the transformative potential of the VPP techniques. Hydrogels like GelMA and PEGDA play a vital role in crafting patches for dynamic organs, ensuring tissue regeneration.^264^ The precision and speed of VPP techniques make them highly desirable in the medical field, ushering in future possibilities for regenerative medicine and TE.

## 5 Conclusions

In conclusion, the advent of VPP in additive manufacturing has catalyzed considerable progress in materials and technology, particularly in the realm of biomedical engineering. VPP’s precision and versatility in fabricating smart polymer-based structures responsive to external stimuli offer promising avenues for various biomedical applications. Throughout this review, we have elucidated the intricate mechanisms and diverse applications of VPP, emphasizing its pivotal role in addressing the evolving demands of biomedicine. From intricate geometries to rapid prototyping and material flexibility, VPP presents unparalleled advantages in the biomedical landscape. Furthermore, the exploration of challenges and future directions, including the utilization of bioabsorbable polymers and bioinks, underscores the ongoing quest for innovation and advancement in tissue engineering, drug delivery, and regenerative medicine. As biomedical research continues to evolve, the integration of advanced printing technologies such as VPP promises to revolutionize patient care and medical treatment modalities. This review serves as a comprehensive guide for biomedical engineers and researchers navigating the dynamic landscape of additive manufacturing in biomedical applications, paving the way for transformative breakthroughs in healthcare.

## List of abbreviations

2PP: two-photon polymerization
3D: three-dimensional
4D: four-dimensional
ABS: acrylonitrile butadiene styrene
AESO: acrylated epoxidized soybean oil
AM: additive manufacturing
AUD: aliphatic urethane diacrylate
BCP: biphasic calcium phosphate
β-TCP: β-tricalcium phosphate
BPA: bisphenol A
BPNS: black phosphorus nanosheet
CAD: computer-aided design
CaP: calcium phosphate
CLIP: continuous liquid interface production
DED: direct energy deposition
DLP: digital light processing
DMD: dynamic mask device
DOX: doxorubicin
FDM: fused deposition modeling
GelMA: gelatin methacryloyl
HAMA: methacrylated hyaluronic acid
HAp: hydroxyapatite
HMSCs: human mesenchymal stem cells
hot SLA/DLP: hot lithography/DLP
HPPA: high-performance polyamides
LCEs: liquid crystal elastomers
LVP: linear volumetric printing
MAPs: magneto-active polymers
MHTs: microscaled hollow tubules
NGCs: nerve guidance conduits
NIR: near-infrared
PμSL: projection microstereolithography
PAM: polyacrylamide
PCLDA: polycaprolactone diacrylate
PDLLA-*co*-TMC: poly­(l-lactide-*co*-trimethylene carbonate)
PDMS-UMA: polydimethylsiloxane urethane methacrylate
PEGDA: poly­(ethylene glycol) diacrylate
PHB: poly­(4-hydroxybutyrate)
PHO: polyhydroxyoctanoate
PLA: polylactic acid
PLA-TMC: poly­(lactic acid-*co*-trimethylene carbonate)
PLLA: poly l-lactic acid
PNIPAM-AAc: poly­(*N*-isopropylacrylamide-*co*-acrylic acid)
PSTS: photolithographic-stereolithographic-tandem printing
PU: polyurethane
SF: silk fibroin
SLA: stereolithography
SMMF: shape-morphing micro fish
SMP: shape-memory polymer Strategy
TBA: tert-butyl acrylate
TCP: β-tricalcium phosphate
TE: tissue engineering
TPO: thermoplastic polyolefins
TVP: tomographic volumetric printing
UV: ultraviolet
VPP: vat photopolymerization
